# Prenatal opioid exposure, and health, social and educational outcomes for school‐aged children: A systematic review

**DOI:** 10.1111/add.70510

**Published:** 2026-07-21

**Authors:** Louise Marryat, Dawood Gashgari, Shona Shinwell, Vicky Mitchell, Catriona Connell, Gale Macleod, Senga Robertson, Anne Whittaker, Alison McFadden

**Affiliations:** ^1^ School of Health and Wellbeing University of Glasgow Glasgow UK; ^2^ School of Health Sciences University of Dundee Dundee UK; ^3^ College of Public Health and Health Informatics Umm Al‐Qura University Mecca Saudi Arabia; ^4^ Salvation Army Centre for Addiction Services and Research University of Stirling Stirling UK; ^5^ Moray House School of Education and Sport University of Edinburgh Edinburgh UK; ^6^ Faculty of Health Sciences and Sport University of Stirling Stirling UK

**Keywords:** adolescent, child development, education, health, mothers, neonatal abstinence syndrome, opioid, pregnancy, prenatal drug exposure, systematic review

## Abstract

**Background and aims:**

Recent decades have seen a rise in the number of pregnancies exposed to opioids. Whilst evidence of the impact of such exposure on pregnancy and infant outcomes is fairly robust, the impact of prenatal opioid exposure (POE) on longer‐term outcomes for children and young people remains unclear. This systematic review aimed to examine comprehensively the evidence of the association between prenatal exposure to illicit opioids or medication for opioid use disorder (MOUD) and school‐age health, social and educational outcomes.

**Methods:**

PubMed, Web of Science, Scopus and CINAHL were searched for papers published between 1 January 2015 and 13 February 2025, with a follow‐up search conducted on 28 October 2025. Grey literature was searched through Google. Eligible studies could be quantitative or qualitative in nature, reporting empirical findings exploring POE (illicit opioids or MOUD) and health, educational or social outcomes at ages 5–18 years. Editorials, case reports, literature reviews, commentaries and conference abstracts, as well as non‐English language papers, were excluded. Records were screened by two authors. Risk of bias was assessed using the Risk Of Bias In Non‐randomised Studies – of Exposure (ROBINS‐E) Tool. Due to heterogeneity within both exposures and outcomes, results were synthesised narratively.

**Results:**

Thirty‐two papers comprising 16 studies were identified. Quality was mixed: 17/32 papers had high/very high risk of bias, primarily stemming from longitudinal attrition and lack of controlling for confounding. Five very high risk papers were excluded, leaving 27 included papers. Overall, 31 104 children exposed to opioids and 5 430 272 controls were included. Papers spanned 10 countries, primarily in North America, Australasia and Europe. Study designs included prospective birth cohort studies (*n* = 10), retrospective administrative data studies (*n* = 11), a combination of those two designs (*n* = 5) and one cross‐sectional study. The most robust evidence of an association with POE was for poorer visual outcomes (e.g. general eye disorders, failure of standard visual assessments and specific impairments including strabismus and nystagmus) (4/4 studies indicating an association) and increased hyperactivity and attention difficulties (3/3 studies reporting an association). A majority of studies (*n* = 7/10) reported an association with increased cognitive difficulties across various measures. Results for physical health and brain structure/functioning were inconclusive. Evidence was based on few studies for each domain, often with low numbers of participants.

**Conclusions:**

Prenatal opioid exposure through illicit opioids or medication for opioid use disorder is associated with visual outcomes, hyperactivity and inattention and cognitive functioning at 5–18 years. Almost all research focusses on 5–12 years, with little evidence available for adolescents.

## INTRODUCTION

For over two decades, increases in opioid use in pregnancy through illicit use or medication for opioid use disorder (MOUD) have been reported across high‐income countries [1, 2]. Prenatal opioid exposure (POE) has been linked to health and developmental problems for children in the early years, namely: increased risk of stillbirth or preterm birth; low birthweight; and increased vulnerability to worsened cognitive, psychomotor, behavioural, visual, attention and executive functioning [3, 4, 5]. Beyond the first few years of a child’s life, evidence is limited. We therefore lack the evidence to inform early interventions to support child development where required. Current systematic reviews have focused either on outcomes in the preschool period [3] or on specific outcome domains (e.g. neurodevelopment domains [6] or ophthalmic domains [7]). A review synthesising 43 articles on the impact of POE on child development at 2–18 years identified 21 studies including children of both preschool age and above, but only seven focused on children above preschool age alone, allowing their specific outcomes to be identified [8]. Similarly to the preschool results, a consistent positive association was found between POE and poorer vision and educational attainment, conduct problems, morbidity and maltreatment. Inconsistent findings were reported in relation to inattention, cognitive development, brain structure, neurologic function and socio‐emotional development [8]. Results contained within the primary studies in both reviews were often not robust, however, and were mainly based on small samples of children: of the 43 studies included in the Arter review, 32 had a sample size of fewer than 100 participants per group [8]. Additionally, many studies suffered from differential attrition, as well as lacking a control group [3].

The development of data infrastructure systems in many developed countries has provided opportunities to follow families longitudinally using linked routine data, without substantial loss to follow‐up [9]. Since Arter’s review [8] was published in 2019 there has been a surge of interest in this field because of the opioid crisis [1], resulting in a substantial volume of new research. This coincided with new methodological tools to deal with this complex topic becoming more widely used, potentially leading to more robust research. Alongside this methodological expansion, the increasing recognition of the families affected by parental substance use, as well as an ambition in many countries to tackle a wider array of harms, has led to new policy focusing on supporting children and families (e.g. in policy: ‘Safe and Supported: The National Framework for Protecting Australia’s Children 2021–2031’ [10] and ‘Scotland’s Drug and alcohol services – improving holistic family support’ [11]). This in turn has been coupled with specific support for parents who use substances, including the Parents Under Pressure programme [12], which has been rolled‐out in Australia, the UK and Ireland, as well as a range of other holistic family support programmes [13]. In combination, these policies and programmes may have led to the better identification of children affected by parental substance use in recent years, as well as potentially mitigating some of the wider impacts associated with child development in the context of parental substance use [14]. Our systematic review aimed to comprehensively synthesise published evidence on the health, education and social outcomes at school age (5–18 years) associated with POE through illicit opioid use or MOUD.

## METHODS

This protocol was registered in the International Prospective Register of Systematic Reviews (PROSPERO) on 12 February 2025 (CRD42025651335). Methods and results are reported following the Preferred Reporting Items for Systematic Reviews and Meta‐Analyses (PRISMA) guidelines [15].

A search strategy was developed through preliminary literature searches and consultation with experts. Additionally, a specialist librarian reviewed a draft search strategy to ensure there were no obvious gaps in the search terms and advised on how to optimise the search for each database. The strategy included strings for opioid exposure in pregnancy, and social (e.g. child removal, criminal justice involvement), educational (e.g. qualifications and absences) and health (both physical and mental health) outcomes at age 5–18 years (for the full search strategy, see Appendix S1). The following databases were searched for peer‐reviewed articles, published from 1 January 2015 to 13 February 2025, with a follow‐up search including articles subsequently published up to 28 October 2025: PubMed, Web of Science, Scopus and CINAHL. Forward citations were scanned for additional articles. The first 10 pages of public‐facing Google was searched to capture grey literature for the same time period. We included articles published from 2015. The search results were imported into Covidence [16], which automatically removed duplicates. Two reviewers screened the titles and abstracts, and then two reviewers screened the full texts (LM and one of AMcF, DG, VM or SS). Disagreements were primarily related to reasons for exclusion and were resolved by discussion. The inter‐rater reliability (IRR) at initial screening ranged from 0.21 to 0.73 (mean = 0.47, indicating moderate agreement). The IRR at the full‐text stage ranged from 0 (based on one article) to 0.58 (based on 34 articles) (mean = 0.26, indicating fair agreement).

Eligibility criteria included having an exposure to illicit opioids, methadone or buprenorphine, or a diagnosis of neonatal abstinence syndrome (NAS) or neonatal opioid withdrawal syndrome (NOWS). Diagnoses of NAS/NOWS are assigned when a baby experiences withdrawal symptoms after birth, most often in cases of *in utero* opioid exposure, although NAS can arise following other exposures [17]. NAS is frequently used as a proxy for POE, particularly where data on actual prenatal exposures are unavailable [18].

Studies were excluded if they examined multiple substances without separating opioid effects, focused on opioids used solely for pain relief or assessed opioid use outside the pregnancy period. Eligible articles reported any child outcome between the ages of 5 and 18 years; studies covering broader age ranges were included only when results for this age group were presented separately. Animal studies and those involving children aged under 5 years or adults were excluded. Only empirical quantitative or qualitative research was included, with qualitative studies accepted for their capacity to qualitatively capture important outcomes (e.g. school experiences). Editorials, case reports, reviews, commentaries and conference abstracts were excluded. Studies from any country were eligible if published in English.

### Data analysis

Data were extracted into Excel by two authors (LM and SS) using a pre‐designed template. Extracted data included the study details (author, year, title), exposure, outcome measures, population/setting, results, strengths and limitations. It was initially anticipated that the quality assessment of articles would be undertaken using the Mixed Methods Appraisal Tool [19]. As no qualitative studies were included in the review, risk‐of‐bias analysis was undertaken using the ROBINS‐E tool for observational studies of exposures, which was better suited to these types of analysis [20]. Following the ROBINS‐E algorithm, articles were deemed to be at ‘Very high risk of bias’ if they made no attempt to control for confounding or if they had a ‘very high risk’ rating in at least one domain. By contrast, articles that made an attempt to control for confounding but did not control for all necessary competing exposures were deemed to be at ‘low risk of bias except for concerns about residual confounding’ (for details, see Appendix S3). Screening was undertaken by LM, with one‐third of studies selected randomly for double screening (undertaken by GM, DG, SS, SR or AW).

Findings were synthesised and presented narratively by type of outcome: cognitive functioning, language development, motor function, externalising behaviours (including conduct problems, hyperactivity and inattention), internalising behaviours (e.g. depression and anxiety), physical health (incorporating growth measures, hospital admissions and health service access, and prescriptions), brain development, dental outcomes and social outcomes (e.g. child removal from birth parents). Meta‐analysis was inappropriate owing to heterogeneity in both the exposure and the outcome measurements.

### Patient and public involvement

This study was informed by discussions with women who use opioids, people who use drugs more broadly, clinicians, social workers and charity professionals supporting these groups. These conversations, which explored challenges related to pregnancy and parenting informed the scope of this review. Although the search strategy itself was not discussed directly, insights from participants, such as perceived neurodevelopmental differences in children with POE and concerns about higher rates of child removal among those diagnosed with NAS, helped refine the focus of the review. These discussions also prompted reflection on the presentation and contextualisation of findings, including the use of non‐stigmatising language.

## RESULTS

**FIGURE 1:**
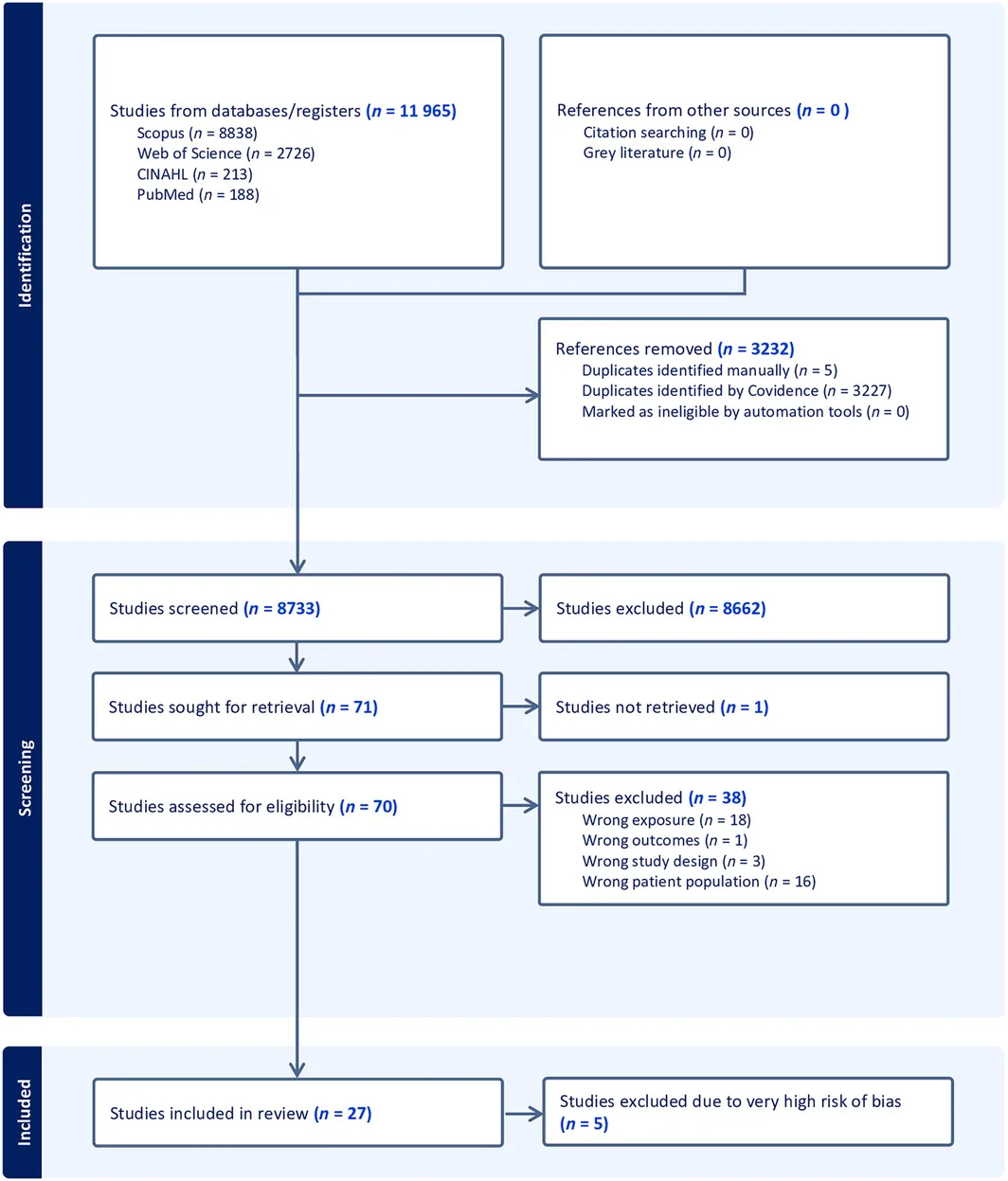
Preferred Reporting Items for Systematic Reviews and Meta‐Analyses (PRISMA) diagram.

The search identified 11 965 articles, 3232 of which were duplicates (Figure 1). No additional articles were identified in the grey literature. Of the 8663 articles screened for title and abstract, 71 full texts were subsequently screened. Thirty‐nine articles were excluded for ineligible exposures (*n* = 18; e.g. broad prenatal drug use, rather than opioid exposure), wrong patient population (*n* = 16; e.g. infants), wrong study design (*n* = 3; e.g. conference abstract), wrong outcomes (*n* = 1; e.g. maternal outcomes) or one full text that was unavailable (despite trying multiple ways to source it, including emailing the author). Thirty‐two articles proceeded to full review, rating and data extraction (Appendix S2). Although some articles used the same population data, we did not group these into studies, as defining a ‘study’ proved challenging, particularly when administrative data from the same population (but with potentially a different method of capturing that population) was used.

Eleven (out of 32) articles were deemed to be of ‘low risk of bias except for concerns about residual confounding’ [21, 22, 23, 24, 25, 26, 27, 28]; four articles had ‘some concerns’ [29, 30, 31, 32]; 12 articles had ‘high risk of bias’ [33, 34, 35, 36, 37, 38, 39, 40, 41, 42, 43]; and five articles had very high risk of bias [44, 45, 46, 47, 48]. Studies with a very high risk of bias typically relied on in‐person follow‐up assessments, resulting in substantial and likely non‐random missing outcome data. Other studies were rated as high risk in this domain owing to poor data linkage or extensive missing administrative data, particularly for the target cohort (e.g. high non‐completion of school assessments [41]). Bias through confounding was common: although most studies attempted to adjust for socio‐economic differences, many over‐adjusted by controlling for variables on the causal pathway (e.g. birthweight, gestation) and often failed to account fully for other teratogens, such as alcohol and nicotine. Bias related to measurement error, post‐exposure interventions and selective reporting was generally low, though only one study was pre‐registered [42]. The five studies deemed at very high risk of bias were excluded, leaving 27 articles for analysis.

These 27 articles comprised 31 104 children exposed to opioids and 5 430 272 controls. All articles were quantitative: designs included prospective birth cohort studies (*n* = 10), retrospective administrative data studies (*n* = 11), a combination of those two designs (*n* = 5) and one cross‐sectional study. Eight articles came from Norway [21, 22, 23, 29, 30, 33, 34, 49], six from the USA [24, 35, 36, 37, 38, 39], four from Australia [25, 40, 41, 50], three from New Zealand [26, 51, 52], two from Canada [31, 32], and one each from the UK [42], Taiwan [43], Israel [27] and Denmark (as part of a wider Scandinavian study) [28]. Most (20/27) articles were published after May 2019 and thus were not included in the Arter review [8].

Eleven articles used NAS as an exposure [24, 25, 31, 32, 36, 37, 38, 39, 40, 41, 50], three used NOWS [21, 22, 33], seven used MOUD (both methadone and buprenorphine) [21, 22, 23, 28, 29, 33, 34], four used MOUD (methadone only) [26, 42, 51, 52], seven used maternal self‐report of opioid use in pregnancy [27, 30, 31, 32, 35, 36, 49] and three used reports of opioid use from administrative data (medical or law enforcement records) [30, 43, 49] (Table 1). Nine articles used multiple ways of ascertaining opioid exposure [21, 22, 30, 31, 32, 33, 36, 47, 49]; for example, eligibility criteria including recorded NOWS and/or MOUD.

**TABLE 1:** Overview of included studies.

Ref.	Article	Country	Data source	Exposure	Number of exposed children	Age at outcome	Outcomes									
							Visual	Motor	Cognitive	Language	Externalising	Internalising	Physical health	Brain	Dental	Social
21	Aslaksen, 2024a	Norway	Administrative data, clinical assessment	NOWS AND MOUD (buprenorphine or methadone)	63	5–13 years		+								
22	Aslaksen, 2024b	Norway	Administrative data, clinical assessment, parent interview	NOWS AND MOUD (buprenorphine or methadone)	63	5–13 years	+									
33	Aslaksen, 2025	Norway	Administrative data, clinical assessment, MRI scanning	NOWS AND MOUD (buprenorphine or methadone)	55	5–13 years	+	+						+		
31	Auger, 2020a	Canada	Administrative data	Maternal self‐report of opioid use or NAS	1791	Max. 12 years	+									
32	Auger, 2020b	Canada	Administrative data	Maternal self‐report of opioid use or NAS	1829	Max. 12 years									+	
35	Bauer, 2020	USA	Administrative data, clinical assessment	Maternal self‐report of opioid use	45	6–15 years			=		=		=			
36	Miller, 2020a	USA	Administrative data, clinical assessment	NAS and/or maternal self‐report of opioid use	233	5 and 10 years					+					
37	Miller, 2020b	USA	Administrative data, clinical assessment	NAS	233	5 and 10 years				**=**						
23	Konijnenberg, 2018	Norway	Administrative data, clinical assessment, including EEG	MOUD (buprenorphine or methadone)	19	9–11 years			**=**					**−**		
29	Konijnenberg, 2020	Norway	Administrative data, clinical assessment, saliva samples	MOUD (buprenorphine or methadone)	19	9–11 years			**+**	**+**		**=**				
34	Konijnenberg, 2021	Norway	Administrative data, clinical assessment	MOUD (buprenorphine or methadone)	20	9–11 years			**−**							
24	Corr, 2021	USA	Administrative data	NAS	17 229	5–10 years							**=**			
42	Hamilton, 2024	Scotland	Administrative data, clinical assessment, case note review	MOUD (methadone)	98	8–10 years	**+**									
51	Jaekel, 2021	New Zealand	Parent interview	MOUD (methadone)	85	9 years					**+**	**+**				
52	Lee, 2019	New Zealand	Parent interview, teacher survey	MOUD (methadone)	85	9.5 years			**+**							
26	Newbury, 2024	New Zealand	Parent interview, clinical assessment	MOUD (methadone)	85	9.5 years				**+**						
38	Lambert, 2024	USA	Administrative data	NAS	371	6–7 years							**=**			
40	Lee, 2024	Australia	Administrative data	NAS	5946	6–15 years^a^							**+**			
41	Oei, 2017	Australia	Administrative data	NAS	Grade 3 = 1663 Grade 5 = 1104 Grade 7 = 499	8–13 years			**+**							
25	Uebel, 2015	Australia	Administrative data	NAS	3842	Up to 13 years							**+**			
50	Uebel, 2024	Australia	Administrative data	NAS	3192	8–15 years			**+**							
43	Lin, 2023	Taiwan	Administrative data	Opioid use in pregnancy (through drug enforcement data)	752 methamphetamine OR opioids (*n* for opioids alone unclear)	7–12 years			**+**		**+**					
27	Ornoy, 2016	Israel	Cross‐sectional survey with clinical assessment	Self‐reported heroin use in pregnancy	158	5–16.5 years			**+**		**+**					
39	Liu, 2019	USA	Administrative data	NAS	1479	6–8 years							**=**			
28	Odsbu, 2023	Denmark	Administrative data	MOUD (buprenorphine or methadone)	130	6 years or older							**=**			
30	Sirnes, 2017	Norway	Administrative data	Maternal self‐report of opioid use or recorded in medical records	16	10–14 years							**=**	**+**		
31	Sirnes, 2018	Norway	Administrative data, MRI scanning	Maternal self‐report of opioid use or recorded in medical records.	11	10–14 years			**+**					**=**		

Abbreviations: EEG = electroencephalogram; MOUD = medication for opioid use disorder; MRI = magnetic resonance imaging; NAS = neonatal abstinence syndrome; NOWS = neonatal opioid withdrawal syndrome; POE = prenatal opioid exposure.

^a^ Also included data for the age band 16–20 years, but we were unable to disaggregate the results for 16–18 years and therefore these have been excluded from the analyses.

Key: 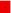, negative relationship, highlighted in red (i.e. POE leads to fewer problems); 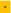, mixed or no overall relationship, highlighted in yellow; 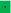, positive relationship, highlighted in green (i.e. POE leads to a greater number of problems).

Regarding outcomes, 11 articles explored cognitive outcomes [23, 27, 29, 34, 35, 41, 43, 44, 49, 50, 52], including educational achievement and delay, eight articles examined physical health [24, 25, 28, 30, 35, 38, 39, 40], six articles studied externalising problems [27, 35, 36, 43, 47, 51] and two articles investigated internalising symptoms [29, 51]. In addition, four articles reported on brain development [22, 23, 30, 49], four articles examined visual outcomes [22, 31, 33, 42], three articles reported language development [26, 29, 37], two articles explored motor outcomes [21, 22], one article investigated dental outcomes [32] and one article reported social outcomes [46], namely child removal. No articles looked at any other social outcomes, such as homelessness or criminal justice system involvement, and notably no articles explored outcomes such as the child’s own substance use. This latter gap may relate to the lack of data in adolescence: no studies focused solely on adolescence, and only three studies detail results for children aged over 13 years [35, 40, 50].

### Visual outcomes

Four articles [21, 22, 33, 42] reported consistently poorer visual outcomes among children with POE. POE was associated with higher risks of general eye disorders, failure of standard visual assessments and specific impairments, including: reduced visual acuity [POE, mean near (binocular) LOGmar = 0.09, SD = 0.11; controls, mean LOGmar = −0.01, SD = 0.11] [22]; markedly higher rates of strabismus (distance and/or near, POE 30.2% vs controls 4.8%) [22] and nystagmus (POE 15.9–20.0% vs controls 0.0–1.6%) [22, 42]; and accommodation amplitude (POE, mean = 12.85, SD = 2.87; controls, mean = 14.24, SD = 2.69) [22]. Children exposed to methadone showed worse outcomes than those exposed to buprenorphine across several domains [22]. One study also found reduced visual–motor integration in children with POE, with deficits that appeared to worsen with age [33].

### Motor skills

Two articles [21, 33] explored motor skills in children aged 5–13 years. Children with POE showed poorer motor functioning across all domains assessed, including manual dexterity, aiming and catching, and balance, with 16% meeting the criteria for definite motor impairment, compared with 0% of controls [21]. Children with POE also demonstrated higher rates of neuromotor difficulties (43% vs 11%) and low visual–motor integration (19% vs 5%) [21]. Among exposed children only, those under 9 years exhibited significantly better motor skills than older children [21]. Hand–eye coordination showed a weak association with reduced right inferior parietal lobule volume in the POE group, though no other brain–motor associations were identified [33].

### Externalising and internalising behaviours

Five articles provided data on externalising behaviours, namely conduct problems and inattention/hyperactivity [27, 35, 36, 43, 51], whilst two articles included an exploration of internalising symptoms, such as depression and anxiety [29, 51]. One article explored these concepts together and found higher levels of difficulties in the opioid exposed group at age 9 years [51].

In terms of broad behavioural problems, the findings were heterogenous: whilst one article found no difference once confounding factors were controlled for [36], other studies found consistently higher levels of behavioural problems among those exposed to opioids [35, 51]. Although to some extent these differences may relate to the different measurements used (e.g. diagnostic data are likely to have a higher threshold vs the direct measurement of behaviours), there may also be issues relating to collider bias and/or small sample sizes masking the true impact of opioid exposure.

All studies exploring hyperactivity/inattention domains found higher levels of difficulties for children exposed to opioids [27, 43, 51]. This included in relation to diagnostic data, where exposed children were more likely to be diagnosed with attention deficit hyperactivity disorder (ADHD) than their unexposed counterparts [27, 43] (e.g. HR 1.43(1.03–1.99) [43]).

Where internalising symptoms were presented separately, the results were mixed: one study found a positive relationship between opioid exposure and emotional difficulties, as assessed with the Strengths and Difficulties Questionnaire (SDQ) (mean difference in score = 1.24, 95% CI = 0.61–1.87) [51]. By contrast, no differences between the exposed and unexposed groups were reported in cortisol levels (a marker of stress) in children aged 9–11 years [29].

### Brain structure and functioning

Four articles investigated associations between POE and brain structure [22, 30] and functioning [22, 23, 30, 49]. All included studies found differences in brain volume [22, 30]. This included reduced intercranial volume [22, 30], with exposed children having, for example, 6.5% reduced basal ganglia, 9.2% smaller caudate, 7.6% smaller thalamus and 10.3% smaller cerebellar [30]. The samples were small overall (POE, *n* = 23–30).

Brain functioning was assessed using electroencephalography (EEG) [22] and functional magnetic resonance imaging (fMRI) [49]. Across studies, no significant differences were observed between POE and control groups in neural amplitudes, activation patterns or latencies [22, 49]. One exception was the finding of faster responses to incongruent rather than congruent trials in the POE group, which the authors suggested may reflect reduced peripheral attention, limiting distraction from external stimuli [22].

### Physical health and health service use

#### Anthropomorphic outcomes

Children with POE were found to have a lower weight than controls: Bauer noted a 2.8 kg difference at age 8 years, rising to a 6.3 kg difference at 15 years [35]. No difference was reported in terms of height [35] or head circumference [30, 35] at school age.

#### Physical health conditions

Limited data were available at school age, impacting the ability to draw conclusions: Lambert found a trend towards higher levels of any diagnosis for children aged 6 years with a history of POE, compared with unexposed children; however, the numbers were too small to show significant differences [38]. One study indicated that children were more likely to receive, by the age of 13 years, a diagnosis of diseases of the digestive system (OR = 1.10, 95% CI = 1.01–1.19), diseases of the skin (OR = 1.14, 95% CI = 1.06–1.24), injuries/poisoning (OR = 1.17, 95% CI = 1.10–1.24), accidents (OR = 1.26, 95% CI = 1.18–1.34) and assaults (OR = 4.20, 95% CI = 3.58–4.93) [37]. Data on asthma was inconclusive, with no effect found in one study [28], but with a positive association reported in another (OR = 1.24, 95% CI = 1.14–1.31) [25].

#### Hospitalisation and health service use

Findings on in‐patient admissions were mixed. Three articles reported higher hospitalisation rates among children with NAS [25, 39, 40] (e.g. 61.6% vs 50% by age 10 years [25]), whereas another found fewer in‐patient visits between the ages of 5 and 10 years [24]. Evidence for emergency department use was also inconsistent: one study reported fewer visits among exposed children [24], while another found higher use at age 6–7 years but lower use at age 8 years, although the numbers were small [39]. The only article reporting outpatient visits showed slightly lower rates for children with NAS at age 5–7 years, but slightly higher rates by age 8 years [24], which authors suggested might reflect higher early preventive care use in this cohort. The length of hospital stay did not differ between groups, but hospital costs were higher for children with POE [40].

#### Prescriptions

Two articles reported higher levels of prescription drug insurance claims at age 5–8 years for children with POE [24, 39], with one study conversely finding lower levels at ages 9 and 10 years [24]. Participant numbers in these latter years were very small, however.

### Dental outcomes

One article examined dental outcomes up to age 12 years and found substantially higher rates of dental caries among children with POE (68.7%, 95% CI = 59.0%–80.1%) compared with controls (27.0%, 95% CI = 27.3%–37.7%) [32]. After adjusting for confounders, POE was associated with an increased risk of any caries (HR = 1.40, 95% CI = 1.11–1.77) and enamel, dentin or cementum caries (HR = 1.47, 95% CI = 1.16–1.87), but not pulp caries (HR = 1.20, 95% CI = 0.94–1.73) [32]. Differences at age 5–7 years were not significant. Notably, only opioid exposure during pregnancy (rather than pre‐pregnancy exposure) was linked to poorer dental outcomes, suggesting the association is specific to POE rather than the postnatal environment [32].

### Social outcomes

One article investigated social outcomes, namely care placements and attachment [46]. It found that 37.1% of children who were living with their birth parent at age 6–8 years had experienced at least one care placement away from home by this age (no control group available). These children were found to have lower levels of attachment difficulties, compared with those removed from their birth parent [46].

### Cognitive and language development

Cognitive development was assessed through global cognitive measures, memory, intelligence quotient (IQ) and educational performance across 10 studies [23, 27, 29, 34, 35, 41, 43, 49, 50, 52]. Seven articles reported poorer cognitive outcomes for children with POE across multiple domains [26, 29, 41, 43, 49, 50, 52], whereas three found no group differences, mixed findings or some advantages for exposed children [23, 34, 35], such as comparable affective decision‐making [34].

Three articles identified significantly lower verbal IQ among exposed children [27, 29, 35], with mixed results for performance and full‐scale IQ: two articles reported lower scores for POE [27, 29], while another found no differences for age 7–15 years [35]. Ornoy reported similarly reduced scores in children with paternal opioid use, suggesting possible genetic or environmental contributions [27]. One article also found increased rates of developmental delay (HR = 1.56, 95% CI = 1.18–2.13) and intellectual disability (HR = 2.37, 95% CI = 1.32–4.25) among exposed children [27]. Memory performance was poorer among children with POE, with deficits observed in memory speed and accuracy [49].

Two articles using teacher‐reported outcomes found consistent educational delays at age 8–13 years in literacy and numeracy [41, 52]. Oei demonstrated a widening achievement gap, with 37.7% of exposed children failing to meet national standards by age 12–13 years compared with 18.4% of controls [41]. More than half of teachers rated children with POE as delayed in reading, maths or writing relative to peers [52].

Three articles explored language development at age 5 years [37] and at age 9–11 years [26, 29, 37]. At age 5 years, no difference in language delay was found between the POE group and an unexposed high‐risk group (81% vs 82% with language delay) [37]. At age 9–10 years, however, all articles reported higher levels of language delay among children with POE, compared with controls [26, 29, 37] (e.g. 24% of exposed children had language delay vs 12% of unexposed children [37]). This was the case across both receptive and expressive language [26].

## DISCUSSION

In the last 10 years, 27 articles (excluding five at very high risk of bias) exploring associations between POE and outcomes at school age have been published, 20 of which have been published since the previous review was conducted [8]. This has significantly broadened the evidence base, allowing the inclusion of newly examined areas such as dental outcomes.

Similarly to outcomes at preschool [3], the strongest evidence appeared to be for poorer visual outcomes, including nystagmus, strabismus and visual acuity, as well as motor–visual integration [22, 31, 33, 42]. New evidence highlighted poorer visual development for children exposed to methadone compared with buprenorphine [33, 42], in line with recent findings at earlier ages [53].

There was strong support for an association between POE and externalising behaviours, particularly in hyperactivity/inattention [27, 43, 51]. Unlike at preschool, this extended at school age to include a higher likelihood of a diagnosis of ADHD [27, 43]. People who use opioids themselves have a far higher prevalence of ADHD [54], *and* as ADHD is a highly heritable condition, around 36%–40% of children with a parent with ADHD received a diagnosis [55]. Therefore, newer research incorporating paternal opioid use suggested that both inherited liability and prenatal exposure contribute to ADHD risk, with evidence that POE may confer additional risk, particularly for girls [27]. Future studies exploring ADHD should incorporate, where possible, data on parental ADHD.

There was also an association between POE and poorer cognitive functioning in school age children, which was not yet apparent at preschool age [3]. This relationship persisted across most measures (including verbal IQ, memory, diagnosed developmental delay or intellectual disability, and in teacher‐rated educational delay), and across age groups, sometimes demonstrating widening inequalities between the groups with age.

New studies offered no further clarity on the relationship between POE and physical health measures and brain structure/functioning. POE had a weak negative association with motor skills, internalising symptoms, and dental and social outcomes. However, evidence was based on few articles, often with small samples. Indeed, evidence remains weak across many areas, reflecting ongoing methodological limitations in studies published since previous reviews, including attrition, measurement heterogeneity and lack of controlling for confounding factors [3, 8]. Despite an increase in large‐scale administrative data and pooled data studies, just 11 articles contained an exposed group with over 100 participants. Many studies suffered from substantial attrition: even in larger studies, by 7 or 8 years of age the outcomes were unreportable owing to small numbers. Having statistical power is critical when attempting to establish rare but important outcomes, such as mortality or serious health conditions [56]. Data from the adolescent period was sparse, limiting evidence for this age group in the domains explored, but also resulting in a dearth of evidence around adolescent‐specific domains, such as the children’s own substance use and adolescent‐onset mental disorders, such as schizophrenia [57]. Part of this problem is likely associated with the lack of high‐quality data on exposures available in historic data. As children age, linkages to education, health and criminal justice data sets may be more informative for understanding outcomes. Outside of Scandinavia, where levels of opioid exposure are low, these linkages are currently limited at a national level, albeit with promising regional‐level results [50].

Exposures remained highly heterogenous in their capture. Just one study explored the impact of different doses of prescribed opioids [42], and none investigated the timing, duration, frequency or route of exposure. Despite more advanced causal inference techniques now being available to examine associations between POE and child outcomes, almost all articles in this review used predictive modelling, rather than more advanced causal inference techniques [58]. Most studies attempted to control for the socio‐demographic characteristics of families in their designs; however, few studies adequately controlled for other competing exposures, including tobacco, alcohol, and other teratogenic and psychotropic drugs. Simultaneously, many studies over‐adjusted models for confounding factors on the causal pathway, such as birthweight.

Alongside improvements to data infrastructure in recent years, policy interest in this topic has increased, which has, in many cases, led to better support for children with POE and their families [13]. Variation in clinical practices and policy responses to opioid use in pregnancy both within and across nations is likely to shape observed health, social and educational outcomes. Countries with greater access to medication for opioid use disorder, less punitive legal frameworks and stronger integration of health, social care and education systems [59] may mitigate longer‐term child vulnerabilities through improved maternal engagement in treatment and continuity of support. Consequently, more recent results from some regions with improved care pathways may show weakened relationships for some outcomes, particularly those that may be highly influenced by the environment in which the child lives. By contrast, fragmented services, inconsistent post‐neonatal follow‐up and more stigmatising policy environments [60] may exacerbate developmental and educational inequalities, contributing to poorer outcomes despite broadly comparable economic resources.

### Implications for research, policy and practice

Despite methodological limitations and heterogeneity in the available evidence, this review has important implications for policy and practice across health, education and social care. While causality cannot be robustly established, the convergence of findings suggests that children with POE represent a group with elevated developmental vulnerability who warrant proportionate, non‐stigmatising attention within universal health systems.

The results highlight the need for consistent, evidence‐informed antenatal support that clearly communicates both known and uncertain longer‐term child outcomes, while continuing to emphasise the importance of treatment engagement and stabilisation through medication for opioid use disorder. Policy frameworks should support balanced discussions that avoid punitive or coercive use of information and situate POE within broader social, genetic and environmental contexts.

The review underscores the limitations of current service models that focus primarily on neonatal outcomes. Emerging evidence of associations with later visual, attentional and cognitive difficulties supports the consideration of longitudinal developmental surveillance extending into school age, embedded within existing universal child health systems, with clear referral pathways. An enhanced awareness of visual difficulties and neurodevelopmental needs may facilitate the earlier identification and intervention, reducing the risk of difficulties becoming entrenched.

Associations with externalising behaviours and ADHD have implications for assessment and support, including the need to consider familial neurodevelopmental risk and sex differences, and to prioritise needs‐based responses to externalising behaviours over interpretations rooted in social adversity alone. Educational settings are likely to play a key role in early identification, supporting preventative rather than responsive approaches.

Overall, while the evidence remains constrained, proportionate early identification and support may help mitigate cumulative disadvantage for this highly vulnerable group of children.

### Limitations of the review

Only articles in English were included, and thus some articles from non‐English speaking countries may have been missed. We included studies from the past 10 years to provide a thorough overview of recent studies (especially with the increasing use of large administrative data), and to capture studies not included in the Arter review [8], which contained articles published up until May 2019. There is an overlap of five articles with Arter [8], and readers should be aware of this if synthesising the two reviews. ROBINS‐E excludes studies where no attempt is made to control confounding. This resulted in the exclusion of one article [48], which contained inconclusive results on unadjusted differences in brain functioning and pain thresholds for children with POE. Four further articles were excluded owing to very high bias in other domains. Even in the short time span examined in this review the types of illicit opioids being used has changed [61], and thus the results may not be applicable to children with different types of POE.

## CONCLUSION

This review synthesises evidence from the past decade on associations between POE and outcomes at school age. It substantially extends the evidence base since the previous review. While research has increased, evidence remains limited by small samples, attrition, heterogeneous exposure definitions, and minimal consideration of dose, timing or duration. Most studies relied on predictive modelling, and evidence relating to adolescence was notably sparse.

Despite these limitations, several findings were consistent. The strongest evidence at school age was for poorer visual development, with stronger associations for methadone than for buprenorphine. Evidence supported associations between POE and externalising behaviours, particularly hyperactivity and inattention, extending to a higher likelihood of ADHD diagnoses. Newer research suggests that both inherited liability and prenatal exposure contribute to ADHD risk, with POE conferring additional risk, particularly for girls. In contrast to preschool findings, associations with poorer cognitive functioning were more consistently observed at school age.

Whilst it was not the aim of this review to establish causality, these findings suggest that vulnerabilities associated with POE may become more apparent with age, underscoring the importance of proportionate, non‐stigmatising surveillance and early support to reduce cumulative disadvantage.

## AUTHOR CONTRIBUTIONS

**Louise Marryat:** Conceptualization; formal analysis; funding acquisition; writing—original draft; writing—review and editing; project administration; methodology. **Dawood Gashgari:** Formal analysis; writing—review and editing. **Shona Shinwell:** Formal analysis; writing—review and editing. **Vicky Mitchell:** Formal analysis; writing—review and editing. **Catriona Connell:** Writing—review and editing; conceptualization. **Gale Macleod:** Conceptualization; writing—review and editing. **Senga Robertson:** Writing—review and editing; methodology. **Anne Whittaker:** Conceptualization; writing—review and editing; funding acquisition. **Alison McFadden:** Conceptualization; writing—review and editing; formal analysis.

## DECLARATION OF INTERESTS

The researchers have no competing interests to declare.

## Supporting information


**Appendix S1.** Detailed search strategy.
**Appendix S2.** Data extraction table.
**Appendix S3.** ROBINS‐E Tool results.

## Data Availability

The data that supports the findings of this study are available in the supplementary material of this article.

## References

[1] Hirai AH , Ko JY , Owens PL , Stocks C , Patrick SW . Neonatal Abstinence Syndrome and Maternal Opioid‐Related Diagnoses in the US, 2010‐2017. JAMA. 2021;325(2):146–155. 10.1001/jama.2020.24991 33433576 PMC7804920

[2] Turner SD , Gomes T , Camacho X , Yao Z , Guttmann A , Mamdani MM , et al. Neonatal opioid withdrawal and antenatal opioid prescribing. CMAJ Open. 2015 Jan;3(1):E55–E61. 10.9778/cmajo.20140065 PMC4382031 25844370

[3] Robertson S , Hughes T , Boardman J , McFadden A , Whittaker A , Marryat L . Impact of exposure to opioids in pregnancy on offspring developmental outcomes in the preschool years: an umbrella review. BMJ Paediatrics Open. 2025;9(1):e003058. 10.1136/bmjpo-2024-003058 39788873 PMC11749434

[4] Leyenaar JK , Schaefer AP , Wasserman JR , Moen EL , O’Malley AJ , Goodman DC . Infant mortality associated with prenatal opioid exposure. JAMA Pediatr. 2021;175(7):706–714. 33843963 10.1001/jamapediatrics.2020.6364 PMC8042571

[5] Graeve R , Balalian AA , Richter M , Kielstein H , Fink A , Martins SS , et al. Infants’ prenatal exposure to opioids and the association with birth outcomes: A systematic review and meta‐analysis. Paediatr Perinat Epidemiol. 2022Jan;36(1):125–143. 10.1111/ppe.12805 34755358

[6] Balalian AA , Graeve R , Richter M , Fink A , Kielstein H , Martins SS , et al. Prenatal exposure to opioids and neurodevelopment in infancy and childhood: A systematic review. Front Pediatr. 2023;11:1071889. 36896405 10.3389/fped.2023.1071889 PMC9989202

[7] Hemmati Z , Conti AA , Baldacchino A . Ophthalmic outcomes in children exposed to opioid maintenance treatment in utero: A systematic review and meta‐analysis. Neurosci Biobehav Rev. 2022;136: 104601. 35263646 10.1016/j.neubiorev.2022.104601

[8] Arter SJ , Tyler B , McAllister J , Kiel E , Güler A , Cameron Hay M . Longitudinal outcomes of children exposed to opioids in‐utero: A systematic review. J Nurs Scholarsh. 2021;53(1):55–64. 10.1111/jnu.12609 33225521

[9] Marryat L , Gajwani R , Graham S , Henderson M , Puckering C , Thompson L , et al. With great (statistical) power comes great responsibility: A comment on the ethics of using administrative data to investigate marginalised populations. Big Data Soc. 2024Dec;11(4):20539517241296058. 10.1177/20539517241296058

[10] Commonwealth of Australia . Safe and Supported: The National Framework for Protecting Australia’s Children 2021–2031 2021. https://www.dss.gov.au/system/files/documents/2024‐10/dess5016‐national‐framework‐protecting‐childrenaccessible.pdf (accessed 10th February 2026).

[11] Scottish Government . Drug and alcohol services ‐ improving holistic family support. 2021. https://www.gov.scot/publications/improving‐holistic‐family‐support‐towards‐whole‐family‐approach‐family‐inclusive‐practice‐drug‐alcohol‐services/ (accessed 10th February 2026).

[12] Barlow J , Sembi S , Parsons H , Kim S , Petrou S , Harnett P , et al. A randomized controlled trial and economic evaluation of the Parents Under Pressure program for parents in substance abuse treatment. Drug Alcohol Depend. 2019;194:184–194. 10.1016/j.drugalcdep.2018.08.044 30447510

[13] Schiff DM , Partridge S , Gummadi NH , Gray JR , Stulac S , Costello E , et al. Caring for families impacted by opioid use: a qualitative analysis of integrated program designs. Acad Pediatr. 2022;22(1):125–136. 10.1016/j.acap.2021.04.016 33901729 PMC8542059

[14] Smith BT , Davidov DD , Gannon M , Groth CP , Kristjansson AL . Parenting through the eyes of mothers with substance use disorder: Implications for treatment and related services. Drug Alcohol Depend. 2025;274:112782. 10.1016/j.drugalcdep.2025.112782 40675008 PMC13390565

[15] Page MJ , McKenzie JE , Bossuyt PM , Boutron I , Hoffmann TC , Mulrow CD , et al. The PRISMA 2020 statement: An updated guideline for reporting systematic reviews. BMJ. 2021; 372. 10.1136/bmj.n71 PMC8005924 33782057

[16] Covidence systematic review software Melbourne, Australia: Veritas Health Innovation.

[17] Kurup U , Merchant N . Neonatal abstinence syndrome: management and current concepts. Paediatr Child Health. 2021;31(1):24–31. 10.1016/j.paed.2020.10.004

[18] Doherty KM , Scott TA , Morad A , Crook T , McNeer E , Lovell KS , et al. Evaluating definitions for neonatal abstinence syndrome. Pediatrics. 2021;147(1). 10.1542/peds.2020-007393 PMC7780959 33268396

[19] Hong QN , Pluye P , Fàbregues S , Bartlett G , Boardman F , Cargo M . Mixed Methods Appraisal Tool (MMAT), version 2018. Registration of Copyright (#1148552) Canadian Intellectual Property Office, Industry Canada; 2018.

[20] Bero L , Chartres N , Diong J , Fabbri A , Ghersi D , Lam J , et al. The risk of bias in observational studies of exposures (ROBINS‐E) tool: Concerns arising from application to observational studies of exposures. Syst Rev. 2018;7(1):242. 30577874 10.1186/s13643-018-0915-2 PMC6302384

[21] Aslaksen AK , Hoem ML , Vikesdal GH , Voie MT , Haugen OH , Skranes J . Children had increased risks of impaired motor and visual‐motor skills after prenatal exposure to opioid maintenance therapy. Acta Paediatr. 2024Jun;113(6):1331–1339. 10.1111/apa.17175 38415880

[22] Aslaksen AK , Vikesdal GH , Voie MT , Rowlands M , Skranes J , Haugen OH . Visual function in Norwegian children aged 5–13 years with prenatal exposure to opioid maintenance therapy: A case–control study. Acta Ophthalmol. 2024Jun;102(4):409–420. 10.1111/aos.15764. 37702266

[23] Konijnenberg C , Jondalen NM , Husby MF , Melinder A . ERP correlates of cognitive control in children prenatally exposed to methadone or buprenorphine. Dev Neuropsychol. 2018;43(7):642–655. 10.1080/87565641.2018.1493592 29979890

[24] Corr TE , Xing X , Liu G . Longitudinal health care utilization of Medicaid‐insured children with a history of neonatal abstinence syndrome. J Pediatr. 2021;233: 82–89. 33545189 10.1016/j.jpeds.2021.01.067

[25] Uebel H , Wright IM , Burns L , Hilder L , Bajuk B , Breen C , et al. Reasons for rehospitalization in children who had neonatal abstinence syndrome. Pediatrics. 2015;136(4):e811–e820. 26371197 10.1542/peds.2014-2767

[26] Newbury J , Sargayoos M , Bora S , Henderson J . Associations between social adversity, caregiver psychological factors, and language outcomes in 9.5‐year‐old children born to women with opioid use disorder. Child Neuropsychol. 2024;30(5):722–737. 10.1080/09297049.2023.2272338 37872777

[27] Ornoy A , Finkel‐Pekarsky V , Peles E , Adelson M , Schreiber S , Ebstein PR . ADHD risk alleles associated with opiate addiction: study of addicted parents and their children. Pediatr Res. 2016Aug;80(2):228–236. 10.1038/pr.2016.78 27064247

[28] Odsbu I , Handal M , Hjellvik V , Hernandez‐Diaz S , Kieler H , Nørgaard M , et al. Prenatal opioid exposure and risk of asthma in childhood: A population‐based study from Denmark, Norway, and Sweden. Front Pharmacol. 2023;14:1056192. 37214456 10.3389/fphar.2023.1056192 PMC10192698

[29] Konijnenberg C , Melinder A . Salivary cortisol levels relate to cognitive performance in children prenatally exposed to methadone or buprenorphine. Dev Psychobiol. 2020Apr;62(3):409–418. 10.1002/dev.21921 31564069

[30] Sirnes E , Oltedal L , Bartsch H , Eide GE , Elgen IB , Aukland SM . Brain morphology in school‐aged children with prenatal opioid exposure: A structural MRI study. Early Hum Dev. 2017;106:33–39. 10.1016/j.earlhumdev.2017.01.009 28187337

[31] Auger N , Low N , Lee G , Ayoub A , Nicolau B . Prenatal substance use disorders and dental caries in children. J Dent Res. 2020a;99(4):395–401. 10.1177/0022034520906820 32091957

[32] Auger N , Rhéaume MA , Low N , Lee GE , Ayoub A , Luu TM . Impact of prenatal exposure to opioids, cocaine, and cannabis on eye disorders in children. J Addict Med. 2020b;14(6):459–466. 31917733 10.1097/ADM.0000000000000621

[33] Aslaksen AK , Bjuland KJ , Hoem ML , Horgen G , Haugen OH , Skranes J , et al. Children had smaller brain volumes and cortical surface areas after prenatal opioid maintenance therapy exposure. Acta Paediatr. 2025Feb;114(2):398–409. 10.1111/apa.17448. 39377497 PMC11706743

[34] Konijnenberg C , Annika M . Affective decision‐making in children prenatally exposed to opioids. Scand J Psychol. 2021;62(4):529–536. 34037260 10.1111/sjop.12743

[35] Bauer CR , Langer J , Lambert‐Brown B , Shankaran S , Bada HS , Lester B , et al. Association of prenatal opiate exposure with youth outcomes assessed from infancy through adolescence. J Perinatol. 2020Jul;40(7):1056–1065. 10.1038/s41372-020-0692-3 32444681

[36] Miller JS , Anderson JG , Erwin PC , Davis SK , Lindley LC . The effects of neonatal abstinence syndrome on language delay from birth to 10 years. J Pediatr Nurs. 2020;51:67–74. 10.1016/j.pedn.2019.12.011 31923742

[37] Miller JS , Anderson JG , Lindley LC . Behavioral development in children with prenatal substance exposure and neonatal abstinence syndrome: Associated factors and implications. J Child Adolesc Psychiatr Nurs. 2020May;33(2):67–76. 10.1111/jcap.12273 32275115

[38] Lambert J , Arter S , Duah H , Xavier T , Sprague JE . Health outcomes in children with prenatal opioid exposure with and without neonatal abstinence syndrome in the first seven years of life: An observational cohort study. J Nurs Scholarsh. 2024Nov;56(6):767–779. 10.1111/jnu.13000 39560001

[39] Liu G , Kong L , Leslie DL , Corr TE . A longitudinal healthcare use profile of children with a history of neonatal abstinence syndrome. J Pediatr. 2019;204:111–117. 10.1016/j.jpeds.2018.08.032 30270164

[40] Lee E , Schofield D , Dronavalli M , Lawler K , Uebel H , Burns L , et al. Health care needs and costs for children exposed to prenatal substance use to adulthood. JAMA Pediatr. 2024;178(9):888–898. 39037833 10.1001/jamapediatrics.2024.2281 PMC11264092

[41] Oei JL , Melhuish E , Uebel H , Azzam N , Breen C , Burns L , et al. Neonatal abstinence syndrome and high school performance. Pediatrics. 2017;139(2):e20162651. 10.1542/peds.2016-2651 28093465

[42] Hamilton R , Mulvihill A , Butler L , Chow A , Irving E , McCulloch DL , et al. Impaired vision in children prenatally exposed to methadone: An observational cohort study. Eye. 2024;38(1):118–126. 10.1038/s41433-023-02644-3 PMC10764882 37402864

[43] Lin CH , Chen MH , Lin WS , Wu SI , Liao YC , Lin YH . A nationwide study of prenatal exposure to illicit drugs and risk of neurodevelopmental disorders and disruptive behavioral disorders. Asian J Psychiatr. 2023;85:103597. 37141844 10.1016/j.ajp.2023.103597

[44] Konijnenberg C , Melinder A . Verbal and nonverbal memory in school‐aged children born to opioid‐dependent mothers. Early Hum Dev. 2022;171:105614. 10.1016/j.earlhumdev.2022.105614 35772183

[45] Sarfi M , Eikemo M , Konijnenberg C . Children born to women in opioid maintenance treatment: A longitudinal study of child behavioral problems and parenting stress. Front Pediatr. 2022;10:1087956. 36619511 10.3389/fped.2022.1087956 PMC9816796

[46] Sarfi M , Eikemo M , Welle‐Strand GK , Muller AE , Lehmann S . Mental health and use of health care services in opioid‐exposed school‐aged children compared to foster children. Eur Child Adolesc Psychiatry. 2022Mar;31(3):495–509. 10.1007/s00787-021-01728-3 33590310 PMC8940845

[47] Azuine RE , Ji Y , Chang HY , Kim Y , Ji H , DiBari J , et al. Prenatal risk factors and perinatal and postnatal outcomes associated with maternal opioid exposure in an urban, low‐income, multiethnic US population. JAMA Netw Open. 2019;2(6):e196405‐. 31251378 10.1001/jamanetworkopen.2019.6405 PMC6604084

[48] van den Bosch GE , Tibboel D , de Graaff JC , El Marroun H , van der Lugt A , White T , et al. Neonatal pain, opioid, and anesthetic exposure; what remains in the human brain after the wheels of time? Front Pediatr. 2022;10:825725. 10.3389/fped.2022.825725 35633952 PMC9132108

[49] Sirnes E , Griffiths ST , Aukland SM , Eide GE , Elgen IB , Gundersen H . Functional MRI in prenatally opioid‐exposed children during a working memory‐selective attention task. Neurotoxicol Teratol. 2018;66: 46–54. 29408607 10.1016/j.ntt.2018.01.010

[50] Uebel H , Dronavalli M , Lawler K , Lee E , Bajuk B , Burns L , et al. School performance in children with prenatal drug exposure and out‐of‐home care in NSW, Australia: A retrospective population‐based cohort study. Lancet Child Adolesc Health. 2024;8(7):500–509. 38897715 10.1016/S2352-4642(24)00076-2

[51] Jaekel J , Kim HM , Lee SJ , Schwartz A , Henderson JM , Woodward LJ . Emotional and behavioral trajectories of 2 to 9 years old children born to opioid‐dependent mothers. Res Child Adolesc Psychopathol. 2021;49(4):443–457. 10.1007/s10802-020-00766-w 33433780 PMC7943531

[52] Lee SJ , Woodward LJ , Henderson JM . Educational achievement at age 9.5 years of children born to mothers maintained on methadone during pregnancy. PLoS ONE. 2019;14(10):e0223685. 31600325 10.1371/journal.pone.0223685 PMC6786534

[53] Bush E , Nidey N , Merhar S , Wexelblatt S , Schwartz T , Weber S , et al. Instrument‐based vision screening and outcomes in young children following prenatal exposure to buprenorphine or methadone: A retrospective cohort study. J AAPOS. 2025;104259. 40712920 10.1016/j.jaapos.2025.104259

[54] van Emmerik‐van Oortmerssen K , van de Glind G , van den Brink W , Smit F , Crunelle CL , Swets M , et al. Prevalence of attention‐deficit hyperactivity disorder in substance use disorder patients: a meta‐analysis and meta‐regression analysis. Drug Alcohol Depend. 2012;122(1–2):11–19. 10.1016/j.drugalcdep.2011.12.007 22209385

[55] Uchida M , DiSalvo M , Walsh D , Biederman J . The heritability of ADHD in children of ADHD parents: A post‐hoc analysis of longitudinal data. J Atten Disord. 2022;27(3):250–257. 10.1177/10870547221136251 36384349 PMC9969349

[56] Mitani AA , Haneuse S . Small Data Challenges of Studying Rare Diseases. JAMA Netw Open. 2020;3(3):e201965. 10.1001/jamanetworkopen.2020.1965 32202640

[57] Jones PB . Adult mental health disorders and their age at onset. Br J Psychiatry. 2013;202(s54):s5–s10. 10.1192/bjp.bp.112.119164 23288502

[58] Pearl J . An introduction to causal inference. Int J Biostat. 2010;6(2):7. 10.2202/1557-4679.1203 PMC2836213 20305706

[59] Handal M , Skurtveit S , Mahic M , Øhman I , Wikner BN , Tjagvad C , et al. Opioid maintenance treatment of pregnant women in the Scandinavian countries. Nordic Stud Alcohol Drugs. 2020;37(3):298–312. 10.1177/1455072520914114 PMC8899263 35308316

[60] Aleksanyan J , Kawachi I , Choi S . Reproductive rights at the US state level and medication access for pregnant women with opioid use. Soc Sci Med. 2025;366:117630. 10.1016/j.socscimed.2024.117630 39721168

[61] Draghmeh K , Gold MS , Fuehrlein B . Epidemiology of Opioid Use, Misuse, and Overdoses. Curr Addict Rep. 2025;12(1):36. 10.1007/s40429-025-00649-4

