# Supplementary material for: Prenatal opioid exposure, and health, social and educational outcomes for school‐aged children: A systematic review
Source: Addiction. 2026 Jul 21;121(10):2633–45. doi: 10.1111/add.70510 (PMC13578869; doi:10.1111/add.70510)
Supplement: Supplementary file 1 [file ADD-121-2633-s001.docx]

# Supplementary Files

## Supplemental File 1 – Detailed search strategy

|  | **Concept** | **Search Terms** |
| --- | --- | --- |
| Exposure | Opioid use | opioid OR opiate OR “opioid dependence” OR “opioid addiction” OR “opioid misuse” OR “opioid abuse” OR “opioid use disorder” OR “opioid-related disorder” OR “prescription opioid misuse” OR “substance use” OR “substance misuse” OR “substance abuse” OR “substance use disorder” OR “opioid replacement therapy” OR “opioid maintenance treatment” OR “opioid agonist therapy” OR methadone OR buprenorphine OR Fentanyl OR heroin OR “neonatal abstinence syndrome” OR “neonatal opioid withdrawal” |
|  | In pregnancy | Pregnan* OR antenatal OR gestation* OR prenatal OR perinatal OR mother* or maternal |
| Outcomes | Health, social and developmental outcomes | health OR "physical health" OR "mental health" OR "longstanding illness" OR disabilit* OR "chronic conditions" OR "chronic illness" OR "congenital conditions" OR multimorbidity OR comorbidity OR respiratory OR cardiac OR dermatolog* OR gastro* OR hepa* OR growth OR obesity OR "brain injury" OR accidents OR injuries OR puberty OR pregnan* OR "Sexually Transmitted Infections" OR STI OR contraception OR abortion OR "self-harm" OR suicide OR mortality OR death OR depression OR anxiety OR stress OR "substance use" OR "eating disorder" OR drugs OR alcohol OR smoking OR tobacco OR vaping OR "health risk behaviour" OR vision OR cancer OR neurodivers* OR autism OR ADHD OR hyperactiv* OR Education OR school OR qualifications OR attainment OR academic OR "school leavers" OR grades OR cognitive OR GPA OR exclusion OR absence OR attendance OR "additional support" OR offend* OR justice OR "youth crime" OR crim* OR court OR police OR prison OR custody OR violen* OR victim* OR bull* OR rape OR "secure unit" OR "secure accommodation" OR "welfare" OR abuse OR maltreatment OR neglect OR "child removal" OR "kinship care" OR "Looked After" OR sex OR relation* OR behaviour OR "Advers* Childhood Experiences" OR trauma OR risk OR homelessness OR employment OR training |
|  | In child age 5-18 | Age 5-18 specified in filters – no separate keywords |
|  |  | |

In filters, set to human only, 5-18 years. Language not filtered in order to quantify how many excluded due to lack of English version.

**Search for PUBMED (results n. = 188)**

(((Pregnan*[Title/Abstract] OR antenatal[Title/Abstract] OR gestation*[Title/Abstract] OR prenatal[Title/Abstract] OR perinatal[Title/Abstract] OR mother*[Title/Abstract] OR maternal[Title/Abstract])) AND (opioid[Title/Abstract] OR opiate[Title/Abstract] OR "opioid dependence"[Title/Abstract] OR "opioid addiction"[Title/Abstract] OR "opioid misuse"[Title/Abstract] OR "opioid abuse"[Title/Abstract] OR "opioid use disorder"[Title/Abstract] OR "opioid-related disorder"[Title/Abstract] OR "prescription opioid misuse"[Title/Abstract] OR "substance use"[Title/Abstract] OR "substance misuse"[Title/Abstract] OR "substance abuse"[Title/Abstract] OR "substance use disorder"[Title/Abstract] OR "opioid replacement therapy"[Title/Abstract] OR "opioid maintenance treatment"[Title/Abstract] OR "opioid agonist therapy"[Title/Abstract] OR methadone[Title/Abstract] OR buprenorphine[Title/Abstract] OR Fentanyl[Title/Abstract] OR heroin[Title/Abstract] OR "neonatal abstinence syndrome"[Title/Abstract] OR "neonatal opioid withdrawal"[Title/Abstract])) AND (health[Title/Abstract] OR "physical health"[Title/Abstract] OR "mental health"[Title/Abstract] OR "longstanding illness"[Title/Abstract] OR vision[Title/Abstract] OR cancer[Title/Abstract] OR neurodivers*[Title/Abstract] OR autism[Title/Abstract] OR ADHD[Title/Abstract] OR hyperactiv*[Title/Abstract] OR Education[Title/Abstract] OR school[Title/Abstract] OR qualifications[Title/Abstract] OR attainment[Title/Abstract] OR academic[Title/Abstract] OR "school leavers"[Title/Abstract] OR grades[Title/Abstract] OR cognitive[Title/Abstract] OR GPA[Title/Abstract] OR exclusion[Title/Abstract] OR absence[Title/Abstract] OR attendance[Title/Abstract] OR "additional support"[Title/Abstract] OR offend*[Title/Abstract] OR justice[Title/Abstract] OR "youth crime"[Title/Abstract] OR crim*[Title/Abstract] OR court[Title/Abstract] OR police[Title/Abstract] OR prison[Title/Abstract] OR custody[Title/Abstract] OR violen*[Title/Abstract] OR victim*[Title/Abstract] OR bull*[Title/Abstract] OR rape[Title/Abstract] OR "secure unit"[Title/Abstract] OR "secure accommodation"[Title/Abstract] OR "welfare"[Title/Abstract] OR abuse[Title/Abstract] OR maltreatment[Title/Abstract] OR neglect[Title/Abstract] OR "child removal"[Title/Abstract] OR "kinship care"[Title/Abstract] OR "Looked After"[Title/Abstract] OR sex[Title/Abstract] OR relation*[Title/Abstract] OR behaviour[Title/Abstract] OR "Advers* Childhood Experiences"[Title/Abstract] OR trauma[Title/Abstract] OR risk[Title/Abstract] OR homelessness[Title/Abstract] OR employment[Title/Abstract] OR training disabilit*[Title/Abstract] OR "chronic conditions"[Title/Abstract] OR "chronic illness"[Title/Abstract] OR "congenital conditions"[Title/Abstract] OR multimorbidity[Title/Abstract] OR comorbidity[Title/Abstract] OR respiratory[Title/Abstract] OR cardiac[Title/Abstract] OR dermatolog*[Title/Abstract] OR gastro*[Title/Abstract] OR hepa*[Title/Abstract] OR growth[Title/Abstract] OR obesity[Title/Abstract] OR "brain injury"[Title/Abstract] OR accidents[Title/Abstract] OR injuries[Title/Abstract] OR puberty[Title/Abstract] OR pregnan*[Title/Abstract] OR "Sexually Transmitted Infections"[Title/Abstract] OR STI[Title/Abstract] OR contraception[Title/Abstract] OR abortion[Title/Abstract] OR "self-harm"[Title/Abstract] OR suicide[Title/Abstract] OR mortality[Title/Abstract] OR death[Title/Abstract] OR depression[Title/Abstract] OR anxiety[Title/Abstract] OR stress[Title/Abstract] OR "substance use"[Title/Abstract] OR "eating disorder"[Title/Abstract] OR drugs[Title/Abstract] OR alcohol[Title/Abstract] OR smoking[Title/Abstract] OR tobacco[Title/Abstract] OR vaping[Title/Abstract] OR "health risk behaviour" OR[Title/Abstract]) Filters: Full text, Classical Article, Clinical Trial, Comparative Study, Controlled Clinical Trial, Equivalence Trial, Evaluation Study, Introductory Journal Article, Observational Study, Technical Report, Twin Study, Humans, Child: birth-18 years, from 2015/1/1 - 2025/10/28

**Web of science (results n..= 2726)**

opioid OR opiate OR “opioid dependence” OR “opioid addiction” OR “opioid misuse” OR “opioid abuse” OR “opioid use disorder” OR “opioid-related disorder” OR “prescription opioid misuse” OR “substance use” OR “substance misuse” OR “substance abuse” OR “substance use disorder” OR “opioid replacement therapy” OR “opioid maintenance treatment” OR “opioid agonist therapy” OR methadone OR buprenorphine OR Fentanyl OR heroin OR “neonatal abstinence syndrome” OR “neonatal opioid withdrawal” (Abstract) and Pregnan* OR antenatal OR gestation* OR prenatal OR perinatal OR mother* or maternal (Abstract) and health OR "physical health" OR "mental health" OR "longstanding illness" OR disabilit* OR "chronic conditions" OR "chronic illness" OR "congenital conditions" OR multimorbidity OR comorbidity OR respiratory OR cardiac OR dermatolog* OR gastro* OR hepa* OR growth OR obesity OR "brain injury" OR accidents OR injuries OR puberty OR pregnan* OR "Sexually Transmitted Infections" OR STI OR contraception OR abortion OR "self-harm" OR suicide OR mortality OR death OR depression OR anxiety OR stress OR "substance use" OR "eating disorder" OR drugs OR alcohol OR smoking OR tobacco OR vaping OR "health risk behaviour" OR vision OR cancer OR neurodivers* OR autism OR ADHD OR hyperactiv* OR Education OR school OR qualifications OR attainment OR academic OR "school leavers" OR grades OR cognitive OR GPA OR exclusion OR absence OR attendance OR "additional support" OR offend* OR justice OR "youth crime" OR crim* OR court OR police OR prison OR custody OR violen* OR victim* OR bull* OR rape OR "secure unit" OR "secure accommodation" OR "welfare" OR abuse OR maltreatment OR neglect OR "child removal" OR "kinship care" OR "Looked After" OR sex OR relation* OR behaviour OR "Advers* Childhood Experiences" OR trauma OR risk OR homelessness OR employment OR training (Abstract) and child* OR adolescen* OR teen* (Abstract)

**SCOPUS (results n.= 8838)**

(TITLE-ABS-KEY(opioid OR opiate OR "opioid dependence" OR "opioid addiction" OR "opioid misuse" OR "opioid abuse" OR "opioid use disorder" OR "opioid-related disorder" OR "prescription opioid misuse" OR "substance use" OR "substance misuse" OR "substance abuse" OR "substance use disorder" OR "opioid replacement therapy" OR "opioid maintenance treatment" OR "opioid agonist therapy" OR methadone OR buprenorphine OR Fentanyl OR heroin OR "neonatal abstinence syndrome" OR "neonatal opioid withdrawal") AND TITLE-ABS-KEY(Pregnan* OR antenatal OR gestation* OR prenatal OR perinatal OR mother* OR maternal) AND TITLE-ABS-KEY(health OR "physical health" OR "mental health" OR "longstanding illness" OR disabilit* OR "chronic conditions" OR "chronic illness" OR "congenital conditions" OR multimorbidity OR comorbidity OR respiratory OR cardiac OR dermatolog* OR gastro* OR hepa* OR growth OR obesity OR "brain injury" OR accidents OR injuries OR puberty OR pregnan* OR "Sexually Transmitted Infections" OR STI OR contraception OR abortion OR "self-harm" OR suicide OR mortality OR death OR depression OR anxiety OR stress OR "substance use" OR "eating disorder" OR drugs OR alcohol OR smoking OR tobacco OR vaping OR "health risk behaviour" OR vision OR cancer OR neurodivers* OR autism OR ADHD OR hyperactiv* OR Education OR school OR qualifications OR attainment OR academic OR "school leavers" OR grades OR cognitive OR GPA OR exclusion OR absence OR attendance OR "additional support" OR offend* OR justice OR "youth crime" OR crim* OR court OR police OR prison OR custody OR violen* OR victim* OR bull* OR rape OR "secure unit" OR "secure accommodation" OR "welfare" OR abuse OR maltreatment OR neglect OR "child removal" OR "kinship care" OR "Looked After" OR sex OR relation* OR behaviour OR "Advers* Childhood Experiences" OR trauma OR risk OR homelessness OR employment OR training)) AND PUBYEAR > 2014 AND PUBYEAR < 2026 AND ( LIMIT-TO ( DOCTYPE,"ar" ) ) AND ( LIMIT-TO ( EXACTKEYWORD,"Human" ) )

**CINAHL (Results n.=213)**

XB ( opioid OR opiate OR “opioid dependence” OR “opioid addiction” OR “opioid misuse” OR “opioid abuse” OR “opioid use disorder” OR “opioid-related disorder” OR “prescription opioid misuse” OR “substance use” OR “substance misuse” OR “substance abuse” OR “substance use disorder” OR “opioid replacement therapy” OR “opioid maintenance treatment” OR “opioid agonist therapy” OR methadone OR buprenorphine OR Fentanyl OR heroin OR “neonatal abstinence syndrome” OR “neonatal opioid withdrawal” ) AND XB ( Pregnan* OR antenatal OR gestation* OR prenatal OR perinatal OR mother* or maternal ) AND XB ( health OR "physical health" OR "mental health" OR "longstanding illness" OR disabilit* OR "chronic conditions" OR "chronic illness" OR "congenital conditions" OR multimorbidity OR comorbidity OR respiratory OR cardiac OR dermatolog* OR gastro* OR hepa* OR growth OR obesity OR "brain injury" OR accidents OR injuries OR puberty OR pregnan* OR "Sexually Transmitted Infections" OR STI OR contraception OR abortion OR "self-harm" OR suicide OR mortality OR death OR depression OR anxiety OR stress OR "substance use" OR "eating disorder" OR drugs OR alcohol OR smoking OR tobacco OR vaping OR "health risk behaviour" OR vision OR cancer OR neurodivers* OR autism OR ADHD OR hyperactiv* OR Education OR school OR qualifications OR attainment OR academic OR "school leavers" OR grades OR cognitive OR GPA OR exclusion OR absence OR attendance OR "additional support" OR offend* OR justice OR "youth crime" OR crim* OR court OR police OR prison OR custody OR violen* OR victim* OR bull* OR rape OR "secure unit" OR "secure accommodation" OR "welfare" OR abuse OR maltreatment OR neglect OR "child removal" OR "kinship care" OR "Looked After" OR sex OR relation* OR behaviour OR "Advers* Childhood Experiences" OR trauma OR risk OR homelessness OR employment OR training )

**Limiters** - Full Text; Publication Date: 20150101-20251031; Research Article; Human; Age Groups: Child: 6-12 years, Adolescent: 13-18 years

**Expanders** - Apply equivalent subjects

**Search modes** - Proximity

**Google (searched first 10 pages)**

("prenatal opioid exposure" OR "in utero opioid exposure" OR "maternal opioid use" OR "opioid use during pregnancy" OR "neonatal abstinence syndrome") AND (child* OR adolescent* OR "school-aged" OR youth) AND ("social outcomes" OR behavio* OR "mental health" OR "educational outcomes" OR "school performance" OR "academic achievement" OR neurodevelopment* OR "health outcomes")

## Supplementary File 2a Data extraction table – Included studies

| Ref | **Author/ year/ title** | **Objectives** | **Country** | **Exposure** | **Participants** | **Study type** | **Outcomes measured** | **Results** | **Strengths** | **Limitations** |  |
| --- | --- | --- | --- | --- | --- | --- | --- | --- | --- | --- | --- |
| 21 | Aslaksen, 2024, Visual function in Norwegian children aged 5–13 years with prenatal exposure to opioid maintenance therapy: A case–control study | Aims were to (1) to examine the long-term effects of prenatal exposure to OMT on various aspects of visual function in a relatively large group of children in a Norwegian setting and (2) to assess possible outcome differences between methadone and buprenorphine exposure. | Norway | NOWS AND Opioid Medication Treatment (OMT) - Buprenorphine or Methadone | 63 children diagnosed either with a confirmed diagnosis of NOWS or ‘Observation for NOWS’, and now aged 5-13 years: OMT-exposed group. Inclusion criteria were age, known prenatal exposure to either methadone or buprenorphine as part of the mothers' OMT and no neonatal disease or birth complication (except for NOWS) affecting the central nervous system. Control group of 63 with no history of OMT or neonatal disease and date and sex-matched to main cohort. Both groups recruited from 2 hospitals in different areas of Norway. | Data collected from medical records for background. Outcomes measured via clinical assessment (eye examination and visual assessment). | Medical history: any glass prescription, use of patching, eye surgery.  Visual acuity: LH or ETDRS chart was used, depending on the child's age.  Orthoptic status: Strabismus was assessed with prism and cover test for near and distance. Data on clinically observed nystagmus, either in primary position or gaze-induced, were included. Binocular function was noted in terms of stereoacuity (TNO-test, Stereo Fly Test or Lang stereotest no. 1). Accommodation and near point of convergence were both tested with a RAF-ruler and recorded as the mean value of three measurements. Refractive state: Autorefraction (Nidek ARK-530A) was done before and after cycloplegia. Eye dominance: Eye dominance was tested for distance and near, using the hole-in-card method (Dolman).  Colour vision: At both the participating centres, the HRR test (Richmond Products, Inc., USA) was applied to test colour vision.  Visual field: Peripheral visual field boundaries were assessed for each eye with Goldmann kinetic perimetry (Haag-Streit AG, Köniz, Switzerland), using object V4e. Anterior segment: The anterior segment of the eye was examined with a slit lamp, especially with regard to congenital anomalies, corneal or lens opacities, and pupil reactions. | **Visual function:** There were statistically significant differences in visual acuity (OMT mean 0.09 (SD0.11) vs controls (-0.01 (0.11), manifest strabismus (distance and/or near OMT 30.2% vs controls 4.8%), nystagmus (OMT 15.9% vs controls 1.6%), and accommodation amplitude (OMT mean 12.85 (SD 2.87) vs controls 14.24 (2.69) ) between the OMT-exposed children and the controls. In addition, there was a tendency towards more astigmatism and hypermetropia in the OMT-exposed group. At the eye examination, 12 (19%) of the OMT children and seven (11%) of the controls wore a habitual glass correction. Four children in the OMT group and one in the control group had been operated for strabismus. According to the definition of amblyopia (interocular difference of two lines or more on a logMAR chart), there were three amblyopic children in both the OMT group and the control group.  There was no significant difference in eye dominance.  Children in the OMT group were more likely to experience colourblindness: in the OMT group, 60 children could be tested for colour vision: 10 showed a mild protanomaly, 1 a moderate protanomaly, and 2 had results indicating poor cooperation. In the control group, there was 1 with a mild protanomaly.  There were no differences between then groups in terms of anterior segments.  Differences mostly persistened when polydrug use was controlled for.  The children exposed to methadone had poorer visual acuity, increased frequency of strabismus for distance fixation (both manifest and latent), and a higher percentage of nystagmus, hypermetropia, and astigmatism compared with the buprenorphine group. | Relatively large sample size for this type of study. The non-responders did not differ from the participants on available background data. Low numbers of women reporting polydrug use and alcohol use. Ophthalmologists and optometrists, doing all the visual assessments, were blinded to which group the children belonged to, thus minimizing any interpretation bias of outcomes. | Three different optometrists did the examinations. This may represent a weakness related to interpersonal differences in the assessments. Although the authors note that they had a detailed, common examination protocol at both study locations and held regular meetings during the patient inclusion period to minimize these problems. Lack of ability to control for smoking exposure due to low prevalence in general population. |  |
| 23 | Aslaksen, 2025, Children had smaller brain volumes and cortical surface areas after prenatal opioid maintenance therapy exposure | Aimed to examine visual function, motor and visual-motor development and brain MRI in children with prenatal OMT-exposure | Norway | NOWS AND Opioid Medication Treatment (OMT) - Buprenorphine or Methadone | 55 children diagnosed either with a confirmed diagnosis of NOWS or ‘Observation for NOWS’, and now aged 5-13 years: OMT-exposed group. Inclusion criteria were age, known prenatal exposure to either methadone or buprenorphine as part of the mothers' OMT and no neonatal disease or birth complication (except for NOWS) affecting the central nervous system. Control group of 59 with no history of OMT or neonatal disease and date and sex-matched to main cohort. Both groups recruited from 2 hospitals in different areas of Norway. | Quantitative study using background data from medical records, postnatal data from a questionnaire combined with interview with caregivers, and outcome data through MRI scanning and image analysis, and clinical assessments of motor and visual function. | Brain morphology image analysis: (namely fifteen volumetric variables: thalamus, hippocampus, amygdala, basal ganglia, lateral ventricle, cerebral and cerebellar grey and white matter, different parts of corpus callosum (anterior, mid anterior, central, mid posterior and posterior part) and intracranial volume. Motor function: Movement Assessment Battery for Children (M-ABC) v2. Visual function: Beery-Buktenica Developmental Test of Visual-Motor Integration (VMI) v6. | **Visual function:** No correlations between brain volume and visual functioning were significantly associated. Note that results of visual function alone are explored in a previous paper [834].  **Motor function:** Poorer outcomes for children in the OMT group in the ABC Manual dexterity test, also reflecting eye-hand coordination and working attention, was weakly correlated to reduced surface area of right inferior parietal lobule (*r* = 0.28, *p* = 0.044). No other correlations between brain volume and motor skills were significantly associated. Note the authors state that results of motors skills alone are explored in a previous paper [834].  **Brain morphology**: Children in the OMT group had significantly smaller mean Intercranial Volume (ICV) adjusted for age and sex, 1407 cm^3^ (CI 95% 1379–1434) versus 1450 cm^3^ (CI 95% 1423–1476) in the control group (*p* = 0.026). The exposed group also had smaller absolute volumes of several brain structures including hippocampus, amygdala, basal ganglia, and anterior and mid-posterior part of corpus callosum. The OMT group had significantly smaller cortical surface area in the left caudal middle frontal gyrus, involving 51% of this gyrus, and in the right inferior parietal lobule, affecting 8% of the lobule, as well as significantly smaller cortical thickness in 16% of the left postcentral gyrus. Further models explored adjusting for being Small for Gestational Age (SGA), polydrug exposure and mother’s level of education:  SGA was a significant regressor for both ICV and basal ganglia volume, with children born SGA having a lower mean ICV (−83.1 cm^3^) and a lower mean basal ganglia volume (−1.2 cm^3^), whilst polydrug use and maternal education had no significant effect. | Relatively large sample size for this type of study. The non-responders did not differ from the participants on available background data. | The authors note that all but 4 OMT mothers and only 2 control mothers smoked during pregnancy so it was not possible to distinguish any additional impact of nicotine exposure. |  |
| 22 | Aslaksen, 2024, Children had increased risks of impaired motor and visual-motor skills after prenatal exposure to opioid maintenance therapy | Aimed to examine: 1) the long-term effect of prenatal exposure to OMT on motor and visual-motor skills and to explore any age effect on outcome in the groups examined; 2) the possible modulating effects of known pre-and perinatal risk factors such as polydrug exposure and SGA status at birth, and the impact of possible postnatal confounders like a mother's education level and age at starting kindergarten; 3) the influence of already reported visual abnormalities on neuromotor function in the OMT group. | Norway | NOWS AND Opioid Medication Treatment (OMT) - Buprenorphine or Methadone | 63 children diagnosed either with a confirmed diagnosis of NOWS or ‘Observation for NOWS’, and now aged 5-13 years: OMT-exposed group. Inclusion criteria were age, known prenatal exposure to either methadone or buprenorphine as part of the mothers' OMT and no neonatal disease or birth complication (except for NOWS) affecting the central nervous system. Control group of 63 with no history of OMT or neonatal disease and date and sex-matched to main cohort. Both groups recruited from 2 hospitals in different areas of Norway. | Quantitative study using background data from medical records, Child Behaviour Checklist (Norwegian version) , postnatal data from a clinical questionnaire combined with interview with caregivers. Outcomes assessed by clinical assessment using standardised measures. | Motor function: Movement Assessment Battery for Children (M-ABC) v2. Visual-motor integration/visual function: Beery-Buktenica Developmental Test of Visual-Motor Integration (VMI) v6. Neuromotor soft signs: Motor Function Neurological Assessment (MFNU) test (assesses higher order motor function: adjustment, coordination, rhythm, fluency, inhibition and muscle tone) | Motor skills: OMT exposed children had significantly lower results across outcome domains. M-ABC test showed significantly lower mean values for total score and 3 component scores (manual dexterity, aiming and catching, and balance) compared to controls. 16% of the exposed children and none in the control group had test results indicating definite motor impairment with an M-ABC total score ≤ 5th percentile. In OMT group only, children <9 years had significantly better motor skills than older children.  Visual-motor integration: 19% percent of the OMT group versus 5% of the controls had low visual-motor integration skills defined as score <80 (9th percentile), OR 4.7 (95% CI 1.3-17.6). Again, in OMT group only, children <9 years had significantly better visual-motor integration than older children.  Neuromotor soft signs: difficulties in 43% of the exposed children versus 11% of controls, with OR 6.1 (95% CI 2.4–15.6)  Impact of other factors (within OMT group):  No impact of polydrug exposure on outcomes. SGA associated with lower visual-motor integration but not motor skills. No gender differences. Children attending kindergarten before the age of 21 months had better motor skills than those attending later, p= 0.03. Child participation in sport activities and mothers' education level did not influence motor and visual-motor skills. | Relatively large sample size for this type of study. The non-responders did not differ from the participants on available background data. Low numbers of women reporting polydrug use and alcohol use. | In one hospital the clinician assessing the children was blinded to their group, but not in the other hospital. Retrospective data collection of alcohol use in pregnancy. Lack of ability to control for smoking exposure due to low prevalence in general population. |  |
| 46 | Auger, 2020, Impact of Prenatal Exposure to Opioids, Cocaine, and Cannabis on Eye Disorders in Children | Objective was to examine the association between prenatal substance exposure and future risk of hospitalisation for eye disorders in children. | Canada | ‘Use of opioids’ declared in pregnancy or NAS | 794,099 infants born in all hospitals in Quebec between 2006 and 2016 – tracked until 18th March 2018. This includes 1,791 exposed to opioids. Preterm infants who developed retinopathy of prematurity were excluded. | Longitudinal administrative data study. | Opthamological diagnosis or procedure during hospital admissions in childhood – categorised by anatomical site and by common childhood pathologies. | **Visual function:** Opioids were found to have greater impact on eye disorders than other substances. Prenatal opioid exposure was 1.48 times risk (HR) of any eye disorder (CI 1.02-2.09) and 3.15 times risk of ocular muscle disorders (1.98-5.01). Note no individual sites were statistically significant, but 4/6 of these contained fewer than 5 events. HRs were adjusted for maternal age, parity, multiple birth, tobacco and alcohol use, maternal comorbidity, sex, preterm birth, low birth weight, infant morbidity, socioeconomic deprivation, place of residence, and time period. | Relatively large sample size. Based on administrative data and largely complete in terms of outcomes data. | Only analysed hospitalisations but not eye disorders treated in community settings. No data on visual evoked potentials. Maternal substance use may be underreported. No information on dose or route. Lacked some confounding data on psychosocial environment, maternal nutrition and ethnicity. Tobacco and alcohol use may also be underreported. |  |
| 47 | Auger, 2020, Prenatal Substance Use Disorders and Dental Caries in Children | Aimed to investigate the association of maternal substance use with risk of dental caries hospitalization in children up to 12 y of age. | Canada | ‘Use of opioids’ declared in pregnancy or Neonatal Abstinence Syndrome (NAS) | 790,758 infants born between 2006 and 2016 in all hospitals of Quebec, Canada followed up to 12 y after birth to identify hospitalizations due to dental caries. Note this study looked at a range of substances in pregnancy, but produced isolated results for 1829 children exposed to opioids. | Longitudinal cohort study using administrative data. | In hospital treatment for dental caries up to age 12 measured. These were identified using diagnostic and procedure codes on the hospital discharge abstract. They classified dental caries based on location: 1) caries localized to the enamel, dentin, or cementum and 2) caries involving the dental pulp (dental caries spreading to the pulp can cause sepsis and tooth loss and is a marker of more advanced disease). | **Dental outcomes:** Incidence rate for dental caries by age 12 of 68.7 (Cis 59.0-80.1) compared with 27.0 (26.7-27.3) with no exposure. Hazard ratio for any caries by age 12 of 1.40 (1.11-1.77), enamel, dentin or cementum: 1.47 (1.16-1.87), pulp: 1.20 (0.94-1.73). Adjusted for age at delivery, parity, mental illness, tobacco use, preterm birth, multiple birth, infant sex, perinatal morbidity, socioeconomic deprivation, rurality and time period. Only opioid use during pregnancy was associated with caries, not pre-pregnancy. Hazard ratios for caries at 5-6 years and 7 years, respectively were not statistically significant. | Relatively large sample size. Based on administrative data and largely complete in terms of outcomes data. | Study lacks information on prescription drug use, potency and frequency of substance consumption, and treatment for substance use disorders. Also lacking data onpost-birth opioid use, so difficult to tell whether impact of exposure in pregnancy is direct or not. The results may underestimate the burden of substance use, because underreporting and polysubstance use are likely. There may be the possibility of residual confounding as study lacked data on adverse childhood experiences, childhood environment, paternal substance use, family history of dental caries, ethnicity, breastfeeding, diet, fluoride supplements, and oral hygiene. The study focused on dental caries that required hospital treatment and cannot confirm that the results generalize to milder caries handled in dental clinics. |  |
|  |  |  |  |  |  |  |  |  |  |  |  |
| 33 | Bauere, 2020, Association of prenatal opiate exposure with youth outcomes assessed from infancy through adolescence | To determine relevant clinical associations with exposure to opiates in utero and not to establish causality. | USA | Maternal self-report of opioid use in pregnancy. | Part of Maternal Lifestyle Study, which explores impact of maternal drug use in pregnancy on child outcomes up to age 15 years. Infants born between May 1993 and May 1995 to women who used opiates during pregnancy (exposed group) were matched to a non-opiate-exposed (nonexposed) group of infants by gestational age, race, and sex within the same institution. Eligibility included women 18 or older with infants inborn, singleton, and with a confirmed gestational age ≤43 weeks. Exclusionary criteria were maternal psychiatric disorders, developmental delay, or language difficulties precluding a valid informed consent. Final sample = 45 opiate exposed (mainly methadone and heroin) and 45 controls. | Longitudinal birth cohort with mix of clinic visits and medical records. | Physical exam including growth. Detailed neurological examination at age 7.  Developmental outcomes: the Wechsler Intelligence Scale for Children III (WISC-III; 45) at 7 years, and the Wechsler Abbreviated Scale of Intelligence (WASI; 46) at 9 and 13 years.  Academic achievement: The Woodcock Johnson Tests of Achievement-III (WJ-III; 47) was administered at ages 7, 8, 10, 12, and 15 years. The WJ-III measures academic performance. Letter-Word Identification and Calculation subtests were done at all time points, Passage Comprehension was administered at ages 8, 10, 12, and 15 years, Dictation and Applied Problems at age 7 years only, Reading Fluency at ages 12 and 15 years, and Writing Fluency at age 15 years only. Performance on subtests yields age and grade equivalents compared to the normative sample.  Behaviour: The Child Behavior Checklist was administered to parents at ages 5, 7, 9, 11, 13, and 15 years to assess child behaviour problems. The Vineland Adaptive Behavior Scales was administered to assess child behavior at ages 6, and 10 years. The VABS assesses functioning across four domains: communication, daily living skills, socialization, and motor skills, and generates a Total Composite score. | **Cognitive outcomes:** On Weschler assessments between 4.5 and 13, exposed children had significantly lower verbal IQ scores (p = 0.0350) than non-exposed children. There were no differences on full or performance scale IQ, or on Woodcock Johnson III scales.  **Externalising behaviours:** Opiate-exposed children had consistently higher scores for behavioural problems on the CBCL. There were no differences on the Vineland (VABS) scores at age 6 or 10.  **Neurologic outcomes:** At 7 years, there were no differences in individual findings nor in the total number of neurologic soft signs (including balance, gait, coordination, finger–thumb opposition, abnormal muscle tone (hypertonia/hypotonia), graphesthesia, stereognosis, sensory extinction, and speech).  **Anthropomorphic outcomes:** opioid exposed children had lower weight than non-opioid exposed: this included 2.8 kg difference at 8 years, and a 6.3 kg difference at 15 years. There was no significant difference in height. There was little difference in head circumference. | Opiate exposure was determined by both maternal admission of use and by toxicology confirmation. Those with concurrent cocaine exposure were removed from the sample. Each developmental area was evaluated on multiple occasions over the entire longitudinal study period. | Definitive documentation of drug exposures throughout pregnancy remains elusive as meconium toxicology only confirms relatively recent usage, resulting in reliance on maternal report. There may be residual confounding. The sample size was small. |  |
| 34 | Miller, 2020, Behavioral development in children with prenatal substance exposure and neonatal abstinence syndrome: Associated factors and implication | The purpose of this study was to examine the effect of prenatal substance exposure and NAS on behavioural development through the age of 10. | USA | NAS and/or self-reported opioid use in pregnancy | This study used data from the Maternal Lifestyle Study (MLS). The MLS was a longitudinal study of maternal drug use. Initial enrolment occurred from May 1993 to May 1995 with longitudinal follow-up until 2011 at four centers: Rhode Island (Providence), Florida (Miami), Tennessee (Memphis), and Michigan (Detroit). Inclusion criteria included drug-exposed dyads (n = 658) group-matched with non-exposed dyads (n = 730). Data were collected at 1.5, 5 and 10 years. 661 children were missing data at one of the timepoints required, and thus were removed, leaving a total sample of 727, of whom 32% met NAS criteria. | Cohort study. | Behavioural development: A member of the original MLS team (licensed healthcare providers trained as research evaluators) performed independent assessments of behaviour at 5, and 10 years. Abnormal behavioural development was defined as the report of the examiner's independent assessment of the child's behavioural development following a thorough review of the child's medical and family history, behavioural assessments, and in‐person assessment. | **Externalising behaviours:** 5 years: Significant group differences in abnormal behavioural development at 5 years were identified between the NAS groups: 29% of the NAS group versus 19% of the no‐NAS group. Of children exposed to opioids alone, 33% have behavioural delay, compared with 17% of none-opioid exposed.  10 years: prenatal exposure to opioids was associated with abnormal behavioural development at 10 years (OR = 0.418, p < .001). | Same as #8203 | High levels of missing data (c.50%) and no description of characteristics of missing cases. The NAS variable was created using the MFNAS tool rather than using an ICD-9 code/diagnosis – and signs of withdrawal were only documented at one timepoint. Exposure was determined by maternal self-report or lab results, so may not give a full picture of substances. Over 75% of NAS cohort and 73% of controls had polysubstance exposure. Some confounders not able to be measured. |  |
| 35 | Miller, 2020, The Effects of Neonatal Abstinence Syndrome on Language Delay from Birth to 10 Years. | The aim of this study was to examine the effects of a history of NAS on language development during the first 10 years of life. | USA | NAS | As for 34. | Cohort study. | At ages 5 and 10 years, language delay was assessed by medical chart review of medical diagnoses relating to language delay. | **Language development:** At 5 years of age, 81% with NAS and 82% without NAS had a language delay. At 10 years of age, 24% with NAS and 12% without NAS had a language delay.  Use of child services decreased the log odds of language delay at 10 years of age (OR −1.12) compared with children who did not use child services. Children who lived with non-biological families had an increase in log odds (0.26), compared with those who live with birth parents.  Prenatal polysubstance exposure (whether opioids were 1 or the 3 is unknown) with 3 or more substances (OR 1.23) significantly predicted language delay at 10 years of age. No other exposures predicted language delay in this sample. | This study has a reasonable sample size across 4 US sites with a matched control group. | Difficult to work out how many of sample were exposed or not to opioids: at least 66% were exposed to opioids only or opioids and cocaine. High levels of missing data (c.50%) and no description of characteristics of missing cases. The NAS variable was created using the MFNAS tool rather than using an ICD-9 code/diagnosis – and signs of withdrawal were only documented at one timepoint. The authors suggest that this might lead to over- or under-exaggeration of NAS. Although the MFNAS is a commonly used tool to identify NAS, there are inherent issues with inter-rater reliability due to lack of standard training on use resulting in potential measurement bias. Exposure was determined by maternal self-report or lab results, so may not give a full picture of substances. There were high levels of tobacco and alcohol exposures in the control group. Language delay was reliant on medical diagnosis, so some children who did not engage with health services around speech and language may be missed. |  |
| 26 | Konijnenberg, 2021, Affective decision-making in children prenatally exposed to opioids | To assess: (1) whether there is a difference in decision-making strategy between children of women in OMT and children not prenatally exposed to drugs; (2) whether the OMT and control group differ in their sensitivity to punishment; and (3) whether the OMT and control group differ in their decision-making speed. | Norway | OMT- Buprenorphine or Methadone | Children born to women in OMT in 2004, with 10 year follow-up during 2016-2017. Two groups were included in the study, one group of children born to women in OMT (recruited through OMT centres and treatment facilities in Norway) and one control group consisting of children from low-risk families whose mother reported having used no alcohol or drugs during pregnancy (recruited through local health care centers in and around the city of Oslo and selected based on corresponding due dates). Sample: 20 children born to women in OMT, and 21 controls aged 9-11 at follow-up. | Prospective longitudinal study of children born to women in OMT. | Child version of Iowa Gambling Task. | **Cognitive development:** Children in the  OMT group performed as well overall as the control group in terms of affective decision making (as  evidenced by similar total net scores). Children in  the OMT group performed better over time throughout the task  (as indicated by higher block net scores during block 4) compared  to the control group. While children in the OMT  group learned to take advantageous doors in the last part of the  task, children in the control group kept choosing the  disadvantageous doors. Results suggest that children  prenatally exposed to opioids are able to consider future  consequences when making decisions. Children in the OMT group did also not select advantageous doors faster than disadvantageous doors, compared with controls. | Direct assessment through validated task. | Small sample and high level of attrition. Control group was mainly mid-high SES families, due to difficulties recruiting low SES families. |  |
| 24 | Konijnenberg, 2018, ERP correlates of cognitive control in children prenatally exposed to methadone or buprenorphine | This study aimed to investigate cognitive control in a group of school-aged children prenatally exposed to methadone or buprenorphine (OMT group). | Norway | OMT - Buprenorphine or Methadone | This is the follow up of a cohort of children of women in OMT from birth in Norway, with pregnancy due date Jan 2005-Jan 2007 and using methadone or buprenorphine in pregnancy. Opioid-dependent group were recruited from OMT centres, with comparison group recruited through local health care centres in and around Oslo. 40 children age 9-11 participated in current study (OMT=19, Comparison=21). Response rate unknown. | Birth cohort study | A modified child-friendly version of the Eriksen flanker task was used to measure cognitive control. EEG was recorded whilst stimuli were presented on a LCD monitor. Event-related brain potentials (ERPs) are commonly used to explore the neural processes involved in cognitive control | Task performance: Results revealed no group differences in reaction time between opioid exposed and non-exposed groups (F(1, 39) = 1.18, p = .29, ƞ2 = .03) or amount of errors made (F(1, 39) = .01, p = .92, ƞ2 < .001).  **Brain development:** ERPs: in terms of amplitudes and increased latencies for the N2 (conflict monitoring), P3 (stimulus evaluation), and LPC (response evaluation and motor inhibition). There was no difference between opioid exposed and non-exposed controls. Contrary to the comparison group, the OMT group did have an atypical N2 response, with faster responses to incongruent than congruent trials. The OMT group did not show the expected interference effect, which suggests that they might have a deficit in peripheral attention that prevents them from being distracted. | Comprehensive cohort – due to small numbers of exposed children and compulsory treatment in Norway. Validated cognitive measures used. | Unclear whether atypical response was due to OMT or other confounding factors. Limited sample size – limited statistical power. |  |
| 25 | Konijnenberg, 2020, Salivary cortisol levels relate to cognitive performance in children prenatally exposed to methadone or buprenorphine | This study examined the effects of prenatal methadone and buprenorphine exposure on HPA activity in childhood, measured by salivary cortisol levels. | Norway | OMT - Buprenorphine or Methadone | This is the follow up of a cohort of children of women in OMT from birth in Norway, with pregnancy due date Jan 2005-Jan 2007 and using methadone or buprenorphine in pregnancy. Opioid-dependent group were recruited from OMT centres, with comparison group recruited through local health care centres in and around Oslo. 76% participation rate. Follow-up at 9-11 years = 20 children of women in OMT and 21 control children. | Birth cohort study | Cognitive functioning was assessed using the Wechsler Abbreviated Scale of Intelligence (WASI). WASI contains four sub‐ tests (vocabulary, similarities, block design, and matrix reasoning) that measure general intellectual functioning for individuals between the ages of 6 and 89 years.  Salivary cortisol was measured through saliva samples collected in the laboratory and assessed by a technician who was bling to group status. | **Cognitive development:** The OMT group scored significantly lower on all four subtests (vocabulary (OMT: M=37.70 (SD 8.66); Controls: M=47.10 (SD6.75), similarities (OMT: M=43.30 (SD 4.78); Controls: M=46.90 (SD 8.67), block design (OMT: M=48.50 (SD 10.12); Controls: M=59.95 (SD 12.12), and matrix reasoning (OMT: M=46.65 (SD 10.12); Controls: M=56.48 (SD 7.41)) and had lower composite verbal IQ (OMT: M=86.00 (SD 7.75); Controls: M=95.00 (SD 11.03), performance IQ (OMT: M=96.35 (SD 13.75); Controls: M=112.57 (SD 15.57), and full scale IQ (OMT: M=89.75 (SD 10.24); Controls: M=105.00 (SD 11.75) scores compared to the comparison group. Results controlled or birth weight and maternal education.  **Internalising symptoms:** No differences were found in cortisol levels or reactivity between the two groups. | Cortisol samples measured in lab, which aids compliance with protocols. Technician assessing samples was blinded to the group membership. | Baseline cortisol levels were collected at the laboratory, not at home. These cortisol levels might have been influenced by various factors, including the child's anticipation to the assessment session. It would have been preferred to sample cortisol from saliva at home, during a stress‐free period, however authors decided against this due to problems with compliance. Sample size was limited, which weakens the statistical power of analysis and affects the study's generalizability. Only 6 of 20 infants in the OMT group did not require medical treatment for NAS at birth. Consequently, the study lacks statistical power to exclude type II errors for the analysis of cortisol levels of children in the OMT group with and without NAS at birth. Prenatal exposure to substances was based mainly on self‐report, which is subject to response and recall bias. |  |
|  |  |  |  |  |  |  |  |  |  |  |  |
|  |  |  |  |  |  |  |  |  |  |  |  |
|  |  |  |  |  |  |  |  |  |  |  |  |
| 36 | Corr, 2021, Longitudinal Health Care Utilization of Medicaid-Insured Children with a History of Neonatal Abstinence Syndrome | Aimed to describe and compare children with a diagnosis of NAS to those without NAS through the eleventh year of life, evaluate the number of health care encounters (inpatient admissions, outpatient evaluations, ED visits) and prescribed medications for children with and without NAS, and investigate the total number of medical claims and associated medical costs for children with NAS vs those without NAS. | USA | NAS | Participants identified across 50 states and District of Colombia through Medicaid insurance records. Children born 2003-2013 with diagnosis of NAS, but not born very or extremely prematurely were included (n=17 229). Control group were on sex, type of residence (urban or rural), region, year of birth, years of follow-up, race, growth restriction, and gestational age to balance for potential confounders (n=17 229). | Administrative data | The primary outcomes of the study were health care utilization, defined by the numbers of claims linked to inpatient hospitalizations, outpatient encounters, ED visits, and prescribed medications, and the costs associated with these health care services. Costs were defined as gross payments to a provider for a service, eligible under the medical plan terms. Costs were adjusted for inflation using the Consumer Price Index for All Urban Consumers: Medical Care and are reported in 2018 US dollars. | **Service use/hospital admissions:** Children with NAS had fewer inpatient claims and fewer emergency department claims each year between ages 5 and 10. In terms of outpatient claims, children with NAS had fewer claims between ages 5 and 7, but more outpatient claims between ages 8 and 10, although differences were small. **Prescriptions:** Prescription drug claims were higher for children with NAS at ages 5-8, but lower at ages 9 and 10. Authors note that the NAS group were more likely to have received preventative care and vaccines in the early years, which may explain some of the differences seen. | Uses Medicaid data, which captures large portions of population of interest. | Only explored through diagnosis of NAS and no information on whether this was opioid related or not. No data on how newborn was treated or observed for NAS, or how the infant’s symptoms were managed. The study suffered from attrition with fewer subjects over time with less than 1% of the total population followed through to the final year. In addition, the earliest subjects in this study were born in 2003, prior to the national recognition of the opioid crisis, and their treatment may have differed substantially from infants and children born later in the study when the importance of comprehensive, family-focused treatment was recognized and promoted. |  |
| 48 | Hamilton, 2024, Impaired vision in children prenatally exposed to methadone: an observational cohort study. | Aimed to compare vision fail outcome rates at age 8-10 years between children exposed to methadone in pregnnancy and comparison children after controlling for confounding variables. | Scotland | Methadone (OMT) | Cohort recruited at birth within one Scottish hospital. Eligible exposed infants were born to MMOD mothers after 36 completed weeks’ gestation without congenital ocular abnormality or significant neonatal illness. Comparison infants were born contemporaneously (2008–10) at the same maternity hospital, matched for completed week of gestation, birthweight (±250 g) and material deprivation. 111 children (77 exposed, 34 comparison) were taken from casenote review alone, with 33 children (21 exposed, 12 comparison) having casenote review as well as clinical assessment. | Birth cohort study with clinical assessment. | A detailed visual assessment was conducted: children were ascribed a ‘pass’ or ‘fail’ result based on pre-determined fail criteria including one or more of: acuity poorer than 0.2 logMAR not attributable to refractive error; manifest but not latent strabismus; nystagmus; abnormal stereovision. Visual assessment pass/fail was the primary outcome measure. To quantify the extent of visual problems a novel, composite ‘visual detriment index’ (VDI) was created for the study as a secondary outcome measure. Severity of fail criteria were scored (nystagmus = 3, strabismus = 2, impaired binocular vision = 2, poor acuity = 2) and summed, giving possible scores 0–9. A higher score was attributed to nystagmus because of its relatively higher detriment to vision and to reflect how uncommon it is in the population at large. For non-attenders, case notes were reviewed to ascertain any record of hospital eye service admissions, or a pass through the universal eye screening programme. | **Visual function:** Children born to MMOD mothers were more likely than comparison children to have a visual ‘fail’ outcome: 56/98 vs 12/46 (unadjusted odds ratio, 3.8 (95% CI 1.8–8.2, *p* = 0.001)). Exposed children were also more likely to have attended or be attending hospital eye services and had higher median VDI. Twenty (20%, 95% CI 13–30%) exposed children had nystagmus, none with an explanatory diagnosis or family history. No comparison child had nystagmus. This remained after tobacco exposure in pregnancy was controlled for. There were no differences in outcomes for children exposed to other drugs in addition to methadone, or for those who received pharmacological treatment for NAS. The exception to this was buprenorphine in addition to methadone, which resulted in higher VDI. Higher dosage of methadone was associated with failing visual assessment and with VDI. There was no difference by sex of child or care status. | 98% response rate at birth. Prenatal drug exposure was determined via comprehensive toxicology (maternal urine, infant urine and meconium), maternal casenote review and confidential interview . Low attrition due to combining use of clinical assessment and universal screening results. |  |  |
| 43 | Jaekel, 2021, Emotional and Behavioral Trajectories of 2 to 9 Years Old Children Born to Opioid-Dependent Mothers | The aim of this study was to characterise the behavioural and emotional trajectories from age 2 to 9 years of a regionally representative sample of prenatally opioid exposed children relative to their non-exposed peers. | New Zealand | OMT - Methadone | Children born 2003-2008 at Christchurch women’s hospital (women recruited during 3^rd^ trimester or at birth). Exclusion criteria included very preterm birth (≤32 weeks), congenital abnormality, HIV, fetal alcohol syndrome, and non-English speaking. 99 mothers receiving MMT/100 infants recruited (85% recruitment rate). Retention to age 9=85%. Comparison group of randomly identified non-exposed mothers (108) and infants (110) recruited, with 90% retention to age 9. | Birth cohort study | Participants were assessed at five time points: late pregnancy/ birth, 18 months, 2, 4.5, and 9 years. Strengths and Difficulties Questionnaire (SDQ) completed by caregivers at 2, 4.5 and 9 years during structured parent interviews. | **Internalising Symptoms and Externalising behaviours:** Opioid-exposed children had higher SDQ scores than non-exposed peers at all childhood assessment. The mean difference in scores widened over time from 2.06 [0.61, 3.52] at age 2 years to 7.13 [5.30, 8.96] at age 9 years. This pattern was the same across all four domains. | Prospective study with good recruitment and retention rate. Those with 2/3 SDQs completed had 3^rd^ SDQ score imputed. Control group. Assessments were rigorous and used internationally accepted, standardized measures. | Relatively small sample so statistical power was limited. Only looked at women in treatment (although authors note that untreated women on opioids are rare in NZ). |  |
| 44 | Lee, 2019, Educational achievement at age 9.5 years of children born to mothers maintained on methadone during pregnancy | This study aimed to a) describe the educational achievement of a cohort of 9.5-year-old methadone exposed (ME) children compared to a regionally representative non-ME group, and b) to assess the extent to which any between-group differences in educational achievement reflected the direct effects of prenatal methadone exposure after adjustment for confounding factors spanning other prenatal substance exposures, infant medical risks, and maternal social background factors | New Zealand | OMT - Methadone | See 43. | See 43. | **Educational achievement at 9.5 years:**  Teacher ratings of achievement: Teachers were asked to rate each child’s achievement across eight curriculum domains including reading, mathematics, written language, spoken language, art, physical education, health, and technology. For each domain, teachers indicated whether the child was: (1) more than 12-months delayed, (2) below average, (3) average, (4) above average, or (5) more than 12-months ahead relative to their classroom peers.  Standardised tests: 6 subtests from the standardized Woodcock Johnson-III Tests of Achievement (WJ-III) Form B, Australian adaptation. The Broad Reading subtests were Letter-Word Identification, Reading Fluency, and Passage Comprehension, to measure children’s reading decoding skills, speed and accuracy, and reading understanding. The Broad Math subtests were Calculation, Math Fluency, and Applied Problem Solving, to measure children’s mathematical computation skills, speed and accuracy in solving basic operations, and problem solving ability.  Educational delay: 1SD below comparison group mean.  Specific learning delay: using scores from standardised tests  Special education use/access, as reported by teachers and caregivers. | **Cognitive development:** Teacher ratings: ME children were significantly more likely to be rated by their classroom teacher as being delayed compared to their non-ME peers in a range of subject areas e.g. 52% ME delayed in reading vs 14% controls, 55% delayed in maths vs 16% controls, 65% written language vs 20% controls. Exception was PE, which was similar across groups.  Standardised tests: ME exposed significantly lower scores across all tests. For reading, ME children scored below non-ME children by 16.4 standard points in Letter-word Identification, 13.2 points in Reading Fluency, and 12.2 points in Passage Comprehension. For mathematics, ME children scored below non-ME children by 16.2 standard points in Calculation, 10.8 points in Math Fluency, and 13.5 points in Applied Problem Solving.  Educational delay: ME children were 4 and 4.5 times more likely than non-ME children to be subject to reading (OR = 6.30 [2.94–13.47]) or mathematics delay (OR = 7.35 [3.36–16.07]). 57% vs. 15% were subject to any educational delay (i.e. reading and/or mathematics delay) (OR = 7.47 [3.71–15.02]).  Special education: ME children were 4 times more likely than non-ME children to be receiving support from a special education service at school (36% vs. 9%, p < .001; OR = 5.56 [2.45–12.59]).  Boys were more likely to experience delays but no interactions with group membership were found. | Sample sizes were low, and the study lacked data on some confounders. | Recruitment and retention rates were high. |  |
| 45 | Newbury, 2024, Associations between social adversity, caregiver psychological factors, and language outcomes in 9.5-year-old children born to women with opioid use disorder | The aims of the study were to describe the language ability of children born to women with OUD using a comprehensive language measure and to examine the associations between prenatal opioid-exposure, social adversity, caregiver psychological factors, and child language outcomes at age 9.5 years. | New Zealand | OMT - Methadone | See 43.  This paper reports the findings for 80 opioid exposed children (85% retention), and 98 controls (89% retention). | See 43. | Language assessed through clinical research assessment. The Clinical Evaluation of Language Fundamentals – Fourth Edition – CELF-4 (Australia and New Zealand Version) was used providing data on 3 subscales – core language, receptive and expressive language. | **Language development:** Opioid exposed children had lower mean scores on the core language index (mean = 88.95 vs. mean = 105.69, p < .001), Expressive Language Index (mean = 91.23 vs. mean = 109.69, p < .001), and Receptive Language Index (mean = 90.49 vs. mean = 102.10, p < .001) relative to controls. While there were statistically significant differences between the groups across all the language indices, the mean standard scores in the OE children were in the normal range, ranging from 88.95 to 91.23. Children exposed to opioids were more likely to be in the low language rating group, compared to controls, across all scales, with this exception of low receptive language.  An unusually high proportion (13%) of opioid exposed children (compared with 1% of the NE children) achieved a core language score in the very low range. The odds for opioid exposed children performing in the very low range on the CLI were 19 times greater than the NE children (OR = 19.58 [2.46–156.14], p = .005). This pattern was seen across both receptive and expressive language. | See 43. | See 43. |  |
| 37 | Lambert, 2024, Health outcomes in children with prenatal opioid exposure with and without neonatal abstinence syndrome in the first seven years of life: An observational cohort study. | The objective of this study was to elucidate the incidence and crude incident rate ratio of health outcomes of children with POE during their first 7 years of life, while distinguishing between those with and without a NAS diagnosis, using a regional maternal infant data hub. | USA | NAS | Data from Maternal and Infant Data Hub covers data from two academic medical centers and one pediatric hospital system in the Midwest region of the US. Children born between 2015 and 2022 with data in the MIDH were included. 312 children were excluded from the analysis because (1) ICD codes were not in the dataset; (2) birthweight was >9000 gm; (3) they received a diagnosis for NAS without evidence of opioid exposure, indicating exposure to substances other than opioids. Remaining children were categorized as belonging to one of three exposure groups: unexposed (n=20,130), exposed without a NAS diagnosis (POE–NAS)(1501), and exposed with a NAS diagnosis (POE + NAS) (371). | Retrospective observational study using administrative data | Health outcomes by age 7: measured using the first 14 ICD-10 CM chapters (A00-N99), which comprise diseases and injury to body systems. | **Physical health outcomes:** In 6^th^ year, trend of POE+NAS having highest levels of diagnoses, followed by POE-NAS, compared with unexposed children. However this was not statistically significant, likely due to the very small numbers involved. In year 7 this could not be ascertained due to small numbers. | This used administrative data so did not rely on caregiver recall. | Numbers of POE+NAS were small, especially by years 6 and 7 of life, thus there was very little statistical power to robustly evidence results. |  |
| 39 | Lee, 2024, Health Care Needs and Costs for Children Exposed to Prenatal Substance Use to Adulthood | This study aimed to investigate hospital admissions and related costs of children with and without NAS. | Australia | NAS | Births between July 1, 2001, and December 31, 2020, in New South Wales (NSW). This included those diagnosed with NAS (*ICD-10-AM* P96.1; n = 5946). Controls had no known substance use exposure (n=1 611 351) | Longitudinal linked administrative data study. | The risk of readmission was defined as any inpatient hospital admission after birth discharge. Hospitalization costs were assigned to each episode using the AR-DRGs–specific mean national public hospital costs, adjusted to 2019 to 2020 Australian dollars and by type of substance use during pregnancy. | **Hospitalisation:** Risk of readmission for children with NAS vs no NAS:   - Age 6-10 years 1.19 (1;12-1.26) - 11-15 years 1.27 (1.18-1.37) - 16-20 years (1.41 (1.30-1.59)   Didn’t have longer length of stay in hospital after 6 years, but did have increased costs. | The validity of probabilistic record linkage was high between maternal hospital and birth records (98.1%). Large sample sizes. | Unknown whether NAS was related to opioid exposure specifically. Unmeasured confounding e.g. environmental characteristics unaccounted for. |  |
| 40 | Oei, 2017, Neonatal abstinence syndrome and high school performance | This study aimed to ascertain the relationship between a diagnosis of NAS at post-birth hospital discharge and school performance in standardised national tests between ages 8 and 15. | Australia | NAS | Children born in New South Wales with a diagnosis of neonatal withdrawal from maternal use of drugs of dependency, corresponding to the ICD-10-AM code P96.1 were selected from the Admitted Patient Data Collection database and compared with matched controls and with other children in NSW. Stillbirths, infants born at 44 weeks’ gestation or of unknown gestational age, and those who died before the first test in 2008 were excluded from analysis. Sample size:  Grade 3: NAS=1663; controls=3251  Grade 5: NAS=1104; controls=2160  Grade 7: NAS=499; controls=992 | Linked administrative data study. | The paper uses data from the National Assessment Program: Literacy and Numeracy (NAPLAN) test which was introduced in 2008 to serve as a compulsory, curriculum-based test for children in all Australian schools. It comprises 5 domains of testing: reading, writing, numeracy, spelling, and grammar/ punctuation. This paper explored NAPLAN scored at age 3 (age 8-9), grade 5 (age 10-11) and grade 7 (age 12-13) | **Cognitive development:** Children with NAS had significantly lower scores than either matched controls or other NSW children in every grade and every domain of literacy and numeracy testing.  At Grade 3, 28.8% of children with NAS did not meet NMS in ≥1 domain (vs 12.3% control and 9.8% other NSW children).  At Grade 5, 36.7% of children with NAS did not meet NMS in ≥1 domain (vs 15.8% control and 13.5% other NSW children).  At Grade 7, 37.7% of children with NAS did not meet NMS in ≥1 domain (vs 18.4% control and 14.5% other NSW children). | Large sample, even at later stages. Used standardised testing for outcomes rather than parent/teacher scores. NAS based on meeting of diagnostic criteria by clinicians. | Linkage rates were 77.6% of controls (similar to whole population), but 75.6% for cohort children. No data on specific drugs used by mothers, or on proportion of exposures involving opioids. |  |
| 41 | Uebel, 2015, Reasons for rehospitalization in children who had neonatal abstinence syndrome | This research aimed to determine the health of children with a diagnosis of NAS in the post-neonatal period, through hospitalisations and mortality. | Australia | NAS | The study used all available linked data from New South Wales between 2000 and 2011. Hospitalisation and mortality data were linked with a minimum of 2 years follow-up and a maximum of 13 years. Children with a diagnosis of NAS (ICD 10 code P96.1) in any episode of care was used to define cohort of children with NAS, vs those without NAS. 3842 children with NAS (5 of whom died before discharge) and 1018421 children without NAS, 1856 of whom died before discharge. | Linked administrative data study | Hospitalisation outcomes: any readmission and specific ICD-10 codes relating to episodes of care, as recorded in health records. | **Hospitalisation:** By age 10 years, the risk of readmission to hospital for children with NAS was 61.6% (95% CIs 59.8-63.7), vs. 50.0% (49.9-50.2).  **Physical health outcomes:** It was noted that children with NAS were more likely to receive an admission for mental and behavioural disorders, including autism (OR 2.6 (95% CIs 2.1-3.2)) and that these admissions were more likely to happen at a later age (mean of 63.4 months for children with NAS vs. 41.3 months for those without).  Children with NAS were particularly more likely to have received, before the age of 13, a diagnosis of*:  Asthma (1.24, 1.14-1.31)  Diseases of digestive system (1.10, 1.01-1.19)  Diseases of skin (1.14, 1.06-1.24)  Injuries, poisoning etc (1.17, 1.10-1.24)  Accidents (1.26, 1.18-1.34)  Assaults (4.20, 3.58-4.93)  *All adjusted for younger mother, smoking, gender, prematurity, socioeconomic status, rurality, Indigenous Australian status.  It should be noted that this was data for all admissions up to age 13. Some of these will have happened before school age. | Very large and comprehensive sample size. Authors believe there is little missing data and that data are missing at random on an individual item basis. Data based on diagnostic codes rather than parental recall of illness/injury. | Exposure to opioids could only be ascertained through NAS coding. There were no data on type of drug used. This is likely to be an underestimation of opioid exposure. Diagnostic codes from hospital admissions may only pick up most severe cases and thus true incidence for some conditions may be under ascertained. We could not isolate results to school-age children and so only looked at cumulative experience by age 10/13. It may be that some of these experiences were isolated to the early years. |  |
| 42 | Uebel, 2024, School performance in children with prenatal drug exposure and out-of-home care in NSW, Australia | This study aimed to explore the impact of prenatal drug exposure(PDE) (with and without NAS) on school outcomes up to age 14-15 years. | Australia | Only data for children with NAS will be extracted. | 3192 children with NAS diagnosis (identified through ICD10 code), 644 with PDE, 897487 with no PDE. Cohort comprised born in New South Wales 2001-2020 and completed 1+ NAPLAN test  between Jan 1, 2008, and June 30, 2021, administered in Year 3 (age 8–9 years), Year 5 (age 10–11 years), Year 7 (age  12–13 years), or Year 9 (age 14–15 years | Retrospective administrative data study | Primary outcome = scoring above or below the National Minimum Standard (NMS) in any test domain (mathematics, language, writing, and spelling) at each year level, comparing the relative risk of scoring below NMS. | Children with PDE (regardless of NAS) were more likely to score beneath NMS at each grade, but there were no differences between children with and without NAS with PDE. Proportions not meeting NMS increased over time across all groups: age 8-9 14.2% with no PDE vs 38.5% with NAS and 40.1% with PDE/no NAS; age 14-15 28.6% no PDE, 67.3% NAS, 68.5% PDE no NAS. This held true in adjusted regression model at age 14-15 (adjusted for primary parent completed Year 12, First Nations Australian child, mother’s remoteness score, socioeconomic decile, mother younger than 20 years, serious maternal mental illness, calendar year of birth). | Large numbers up to age 14-15 and excellent linkage rates. Children with PDE more likely to be missing NAPLAN data (20.9% of PDE group missing at year 9 vs 6.1% in no PDE group (no breakdown for NAS group). Assumed to be non-random. Added into ‘fail’ group based on previous evidence. | Unable to tell exact exposure (e.g. methadone vs cocaine), dosage or timing of exposure. Numbers in PDE without NAS group are relatively small. |  |
| 50 | Lin, 2023, A nationwide study of prenatal exposure to illicit drugs and risk of neurodevelopmental disorders and disruptive behavioral disorders | The aim of this study was to investigate the association between prenatal illicit drug exposure and the development of neurodevelopmental disorders and disruptive behavioural disorders (DBD) in children aged 7–12 years. | Taiwan | Opioid use in pregnancy (through drug enforcement data) | Births 2004-2009 in Taiwan. Linked data used to identify illicit drug use among pregnant women: 752 of whom used methamphetamine or opioids. Randomly selected 7520 non-illicit drug users on a 10:1 ration based on maternal age, income and year of childbirth. Results were broken down by opioid vs methamphetamine use, however the numbers in each group were not published. | Linked administrative data study. | Diagnoses recorded in health insurance records of developmental delay, intellectual disability (mild, moderate or severe), ADHD, or DBD. | **Externalising behaviours/cognitive outcomes:** Illicit opioid exposed associated with higher risk of 3 types of neurodevelopmental disorders, but not DBD. Adjusted* hazard ratios for illicit opioid exposure and:   - Developmental delay 1.59 (1.18-2.13) - Intellectual disability 2.37 (1.32-4.25) - ADHD 1.43 (1.03-1.99)   *Models adjusted for maternal age, income, gestational age, maternal comorbidity, neonatal gender, neonatal comorbidity, and maternal medication use (antidepressants and sedative hypnotic drugs). | Exposure data from drug enforcement data and urine toxicology test so no reliance on recall. Diagnoses were made by | Likely to be unmeasured confounding, e.g. infection in pregnancy. Prevalence of neurodevelopmental disorders may be underestimated as only those with diagnoses recorded here. |  |
| 51 | Ornoy, 2016, ADHD risk alleles associated with opiate addiction: Study of addicted parents and their children | This paper aimed to investigate the relationship between genetic (alleles considered to be risk factors for ADHD and for opiate dependency) and environmental (intrauterine exposure to opiates) factors in children born to opiate-addicted mothers and/or fathers, with or without ADHD comparing the molecular findings between the children and their parents. | Israel | Self-reported heroin use in pregnancy | 158 children (64 with a mother or father with heroin dependency during pregnancy and 94 controls with no opiate dependency among parents in pregnancy). Ages of children at outcomes assessment ranged from 5 to 16.5 years. | Cross-sectional survey with clinical assessment | General intelligence: measured using the Wechsler Intelligence Scales for Children.  Behaviour problems: measured through parent-complete Short Child Behavior Checklist (Short Form Assessment for Children (SAC)).  ADHD: Parent-complete Conners Rating Scales for Assessing Attention Deficit and Hyperactivity Revised. The study used scales measuring attention span, hyperactivity, impulsivity, anxiety, and general ADHD measure.  Genetic Studies: DNA samples collected through mouth wash collection. | **Cognitive development:** Wechsler: Intelligence results showed that children with opioid dependent parents were more likely to sit below the general population level of intelligence. There were no differences dependent on whether the mother or father was opiate dependent.  **Externalising behaviours:** A higher rate of ADHD was reported among the children of opiate-dependent mothers (31.3% boys and 12.5% girls, according to parent report) compared to those of the opiate-dependent fathers (15.4% boys and 0 girls). Results were similar on Connor’s scale (56.5% girls with opiate dependent mother and 46.7% boys, vs. 25% girls with opiate dependent father and 46.2% boys). Only girls results were statistically significant.  **Internalising symptoms and externalising behaviours:** CBC:SAC - externalizing and internalizing scores of the children born to opiate-dependent mothers were higher than in those born to addicted fathers, but the differences were not statistically significant.  Genetic risk: there is generally a high rate of carrier state in children whose mothers or fathers are carriers of the different risk alleles for ADHD compared to their carrier state if the parent is not a carrier.  Conclude that maternal opiate exposure increases risk and severity of ADHD, particularly in girls. The lack of a genetic relationship between ADHD in mothers and girls indicates that this is likely due to the exposure to heroin in pregnancy, rather than genetic risk. When both parents used heroin in pregnancy, the rate of ADHD in the offspring rose to 80% with increased severity, which authors theorise is as a result of the genetic contribution from both parents. Although they note an additional possibility is that the very high rate of ADHD is related to postnatal environmental impact. |  |  |  |
| 38 | Liu, 2019, A Longitudinal Healthcare Use Profile of Children with a History of Neonatal Abstinence Syndrome | This study aimed to describe and compare children with NAS with those without NAS through age 8 years, evaluate the number of healthcare encounters (inpatient hospital stays, outpatient evaluations, emergency department [ED] visits) and prescribed medications for children with NAS compared with those without NAS, and evaluate the total number of medical claims and associated medical costs for children with NAS vs those without NAS. | USA | NAS | This study used the MarketScan Commercial Claims and Encounters database which comprises reimbursed healthcare claims from a selection of large employers and commercial health plans across the USA. Data from the years 2005 to 2014 were used to identify children between the ages of 0 and 8 years with initial enrollment during their year of birth. Children with NAS were identified using the ICD-9 code 779.5 (drug withdrawal syndrome in newborn). 1479 had a diagnosis of NAS; 10% of children without NAS were randomly selected as the controls (n = 298 330). | Linked administrative data study. | Primary outcomes were healthcare use, as defined by the numbers of claims related to inpatient hospitalizations, outpatient encounters, ED visits, and medication prescriptions, and costs associated with these healthcare services. | **Service use/hospitalisation:** We extracted findings from ages 6-8: children with NAS had consistently high numbers of claims overall. The had higher levels of outpatient claims at ages 6-8. At ages 6 and 7 children with NAS had slightly higher levels of inpatient claims however there were no claims recorded for children with NAS at age 8. Children with NAS also had higher levels of ED claims at ages 6 and 7, but slightly lower levels at age 8. Children with NAS had higher levels of prescription claims at all ages and higher costs at all ages, but substantially higher at ages 6 and 7. | Large administrative data study based across multiple US states/healthcare sites. Uses diagnostic data so not reliant of parental recall. | No data on specific opioid use, or severity/treatment of NAS. Relies on accurate diagnostic coding. Only picks up children who attended services. Only examined children with diagnosis of NAS covered by private insurance – by contrast >75% children with NAS are covered by Medicaid. Relatively short follow-up period due to discontinuation of health insurance or censoring at the end of study period – leading to smaller number of 6-8 year olds, which I our population of interest. |  |
| 52 | Odsbu, 2023, Prenatal opioid exposure and risk of asthma in childhood: a population-based study from Denmark, Norway, and Sweden | This study aimed to investigate the association between prenatal opioid exposure and childhood asthma in live-born children from Denmark, Norway, and Sweden. | Denmark, Norway, and Sweden | OMT – methadone or buprenorphine | Data were retrieved from the national health- and population registers in Denmark, Norway, and Sweden. A cohort of children born in Denmark 1997–2015, Norway 2005–2015, or Sweden July 1 2006–2013, and whose mothers used opioids pre-pregnancy was identified from the medical birth registers and prescription registers. A total of 1,471,406 children born in Norway/Sweden and 1,207,989 children born in Denmark were eligible for inclusion in the study. There were 14,764 children in the opioid analgesics cohort and 1,595 children in the OMT cohort. Data broken down for older children, however, were only available for the OMT group in Denmark (130 OMT exposed children and 358 unexposed). | Administrative data study. | Asthma was defined based on filled prescriptions of asthma drugs from the prescription registers and/or asthma diagnoses from the patient registers. In children 1 year of age or older, asthma was defined as two or more dispensed inhaled corticosteroids (ATC code R03BA), leukotriene receptor antagonists (R03DC), or fixed combinations of β2-agonists and corticosteroids (R03AK) within 365 days (and not on the same day) during follow-up time, or an asthma diagnosis (ICD-10 J45 or J46) as a primary or secondary diagnosis from specialist healthcare. | **Physical health outcomes:** No association between prenatal exposure to opioids and risk of childhood asthma was found. For children aged 6+ in Denmark (for whom data broken down by age were available), the unadjusted HR was 0.84 (0.34-2.08), with the adjusted 0.94 (0.32-2.80), and so were highly imprecise and unable to reach a conclusion at this age. | Data included all live births in Denmark, Sweden and Norway. Use of propensity scores to balance several confounders across cohorts. | Potential for some OMT prescriptions provided in specialist settings to not be captured in the data. Identifying asthma in children is challenging since symptoms of respiratory infections could be misinterpreted as asthma and antiasthmatic medications could be used to relieve such symptoms. |  |
| 30 | Sirnes, 2017, Brain morphology in school-aged children with prenatal opioid exposure: A structural MRI study | This study aimed to investigate brain morphology in a sample of prenatally opioid-exposed children using magnetic resonance imaging (MRI). | Norway | Prenatal opioid exposure through self-reported opioid use in pregnancy or recorded in medical records | Same sampling as #6258. The final sample for this study consisted of 16 children exposed to opioids in utero and 16 sex- and age-matched controls. | Same as #6258 | MRI scanning was performed, without sedation. Total Intercranial Volume (ICV) was measured using the Buckner et al method. Volumes were calculated. Somatic growth parameters (height, weight, and head circumference) were obtained prior to MRI scanning. | **Anthropomorphic measurements:** Somatic growth parameters, including head circumference, did not differ between the two groups.  **Brain development:** The combined volume of the basal ganglia (accumbens + caudate + putamen + pallidum) was significantly reduced in the opioid-exposed group (adjusted for estimated ICV and birth weight). Relative to controls, exposed children had 6.5% smaller basal ganglia, 9.2% smaller caudate, 7.6% smaller thalamus, and 10.3% smaller cerebellar white matter (differences calculated from estimated marginal means). Group differences were larger after additionally adjusting for ADHD. When researchers attempted to separate results by opioid-only exposure and opioid+ exposure, results were not statistically significant. | Same as #6258  All MRI scans were examined by an experience pediatric neuroradiologist. | Same as #6258 |  |
| 31 | Sirnes, 2018, Functional MRI in prenatally opioid-exposed children during a working memory-selective attention task | The study aims to investigate brain activation patterns in school-aged children with prenatal opioid exposure using functional magnetic resonance imaging (fMRI). | Norway | Prenatal opioid exposure through self-reported opioid use in pregnancy or recorded in medical records. | The opioid-exposed group was derived from a larger group of children with prenatal drug exposure referred to the paediatric department at Haukeland University Hospital in Bergen, Norway, between 1997 and 2012. 20 children had been exposed to opioids, either through heroin or OMT. For each drug-exposed child, the first child of the same gender born at Haukeland University Hospital on the same date, with a birth weight above the 10th percentile (≥3000 g), was invited to serve as a matched control. If they declined, the next child born on the same date (or the nearest date) was contacted. For the 20 opioid-exposed children included, only 17 controls were successfully recruited, hence 20 exposed children and 17 control children, aged 10–14 years, underwent MRI-scanning. 8 children (5 opioid-exposed and 3 controls) were excluded from analyses due to abortion of the fMRI protocol by the child. In addition, scans from 2 opioid-exposed children and 2 controls had to be excluded due to head movement artifacts (> 5 mm translation in any of the 4 experimental conditions) and scans from 2 opioid-exposed children were excluded due to dental braces distorting the images. The final sample therefore consisted of 11 prenatally opioid-exposed children and 12 unexposed controls. | Birth cohort with MRI scanning. | A working memory-selective attention task combining the n-back task and the Stroop colour word task was used. Structural and functional images were acquired through MRI scanning. | **Cognitive development:** Task performance: Overall, mean response accuracies of 86.7% (SD: 7.85%) in the opioid-exposed and 92.5% (SD: 7.89%) in controls were found, when the results from all the four experimental conditions were combined. The opioid-exposed group responded slower, with fewer correct answers than the control group (unadjusted hazard ratio (HR) of control vs. exposed = 1.46 (95% CI: 1.04 to 2.06; p = 0.030)).  **Brain development:** MRI: Results showed increased BOLD activation in prefrontal cortical areas in the exposed group, as compared to unexposed controls, during the most cognitive demanding versions of a working memory-selective attention task. In both groups, task related activation patterns included parietal and prefrontal cortical areas previously shown to be important in working memory and selective attention tasks. There were no areas where the control group showed increased activation relative to exposed children in any of the four experimental conditions. | This study was able to isolate results to children exposed to opioids, as opposed to prenatal drugs more generally. Standardised and validated approaches were used, both in terms of assessments and scanning processes. | Relatively small numbers in the sample which may affect power to detect differences. There may be differences between the two groups which were not accounted for. All opioid exposed children lived in adoptive or foster care in this study and most had been in these placements since an early age. Most children were exposed to multiple drugs and authors were not able to control for other drugs in the research. fMRI uses level of oxygenated blood as a proxy for measuring the activity of neurons. The extent to which differences in the BOLD signal between the groups represent actual differences in neuronal activation as compared to other possible underlying mechanisms, like altered vascularization, is therefore unknown. |  |
|  |  |  |  |  |  |  |  |  |  |  |  |

## Supplementary File 2b Data extraction table – Studies excluded following risk of bias assessment

| **Ref** | **Author/ year/ title** | **Objectives** | **Country** | **Exposure** | **Participants** | **Study type** | **Outcomes measured** | **Result** | **Strengths** | **Limitations** |
| --- | --- | --- | --- | --- | --- | --- | --- | --- | --- | --- |
| 32 | Azuine, 2019, Prenatal Risk Factors and Perinatal and Postnatal Outcomes Associated With Maternal Opioid Exposure in an Urban, Low-Income, Multiethnic US Population | (Among other objectives which are no relevant to the current review) this study aimed to explore the postnatal physical health and neurodevelopmental outcomes of children with opioid exposure compared with children without exposure. | USA | ‘Use of opioids’ self-reported in pregnancy or NAS | Eligible mothers were those who delivered singleton live births at Boston Medical Center (BMC), Boston, Massachusetts, starting in 1998. The enrollment targeted singleton, live-preterm-birth, low birth weight infants, and full term infants with oversampling of preterm/low weight babies. 3153 who continued to receive care at Boston Medical Centre were enrolled in postnatal follow up. Among 454 mothers with opioid exposure, 348 (76.7%) reported heroin use, and 204 (44.9%) reported oxycodone use. | Birth cohort study – mix of medical records and parental interview. | Postnatal physical health and neurodevelopmental disabilities were defined according to ICD-9 and ICD-10 codes from study children’s EMRs. They included being ever diagnosed with attentiondeficit/hyperactivity disorder (ADHD) (ICD-9: 314.0-314.9; ICD-10: F90.0-F90.9), conduct disorder/ emotional disturbance (ICD-9: 312.0-312.9, 313.0-313.9; ICD-10: F91.0-F91.9, F93.0, F93.8, F93.9), and lack of expected normal physiological development (ICD-9: 783.40-783.43; ICD-10: R62.0, R62.50-R62.59). | **Externalising behaviours:** Among children with follow-up records after age 6 years, children exposed to opioids had a higher percentage of diagnoses of ADHD (OR, 2.55; 95% CI, 1.42-4.57) compared with their counterparts.  Children exposed to opioids had higher levels of Conduct problems in unadjusted models (OR 2.00, 95% Cis 1.11-3.59) but this did not stay significant when adjusted for maternal age, household income, race/ethnicity, marital status, maternal education, preterm birth, and low birth weight. There was no significant difference in adjusted or unadjusted models for lack of expected normal physiological development. | The prospective birth cohort design of the study made it possible to investigate early life factors before the onset of opioid-associated NAS and the long-term postnatal outcomes of maternal opioid exposure. BBC is among the largest contemporary prospective US birth cohorts. The study used physician diagnoses extracted from EMRs to identify neurodevelopmental outcomes. This type of data is more reliable than maternal self-reported outcomes, which were used by many prior studies. The study sample is mainly from a low-income, urban, minority population in the Boston area. This is a high-risk yet understudied population. | Data may be subject to exposure misclassification. The authors defined exposure heterogeneously to include illicit, therapeutic, and unknown opioid exposures leading to NAS. This heterogeneity in exposure could lead to differential effects on long-term child outcomes, which could not be disentangled. There may be unmeasured confounding. The degree to which participants lost to follow-up may have resulted in an underestimation of the diagnoses of postnatal outcomes for children exposed to opioids is unknown. Further, this study mainly consisted of a low-income, urban, minority sample, which limits the generalizability of findings. |
| 27 | Konijnenberg, 2022, Verbal and nonverbal memory in school-aged children born to opioid-dependent mothers | The main aim of the study was to investigate memory function in school-aged children of women enrolled in OMT during pregnancy. | Norway | OMT - Buprenorphine or Methadone | This is the follow up of a cohort of children of women in OMT from birth in Norway, with pregnancy due date Jan 2005-Jan 2007 and using methadone or buprenorphine in pregnancy. Opioid-dependent group were recruited from OMT centres, with comparison group recruited through local health care centres in and around Oslo. 76% participation rate. Follow-up at 9-11 years = 20 children of women in OMT and 21 control children. | Birth cohort study | Memory functioning was assessed using four subtests of TOMAL-2; an age-normed memory test designed for individuals between the ages of 5–60 years. To measure non-verbal memory, the subtests Facial Memory and Abstract Visual Memory were used, while verbal memory was assessed using the subtests Word Selective Reminding and Paired Recall. | **Cognitive development**: A statistically significant difference in memory performance was found between the OMT and comparison group ( F(4,36) = 5.37, η2 = 0.37). The OMT group scored lower on both the two non-verbal and verbal tests. The relationship between prenatal methadone/buprenorphine exposure and children's memory performance remained significant after controlling for general IQ, except for Abstract Visual Memory, which no longer was associated with group status after controlling for general IQ. unadjusted model accounted for a significant amount of the overall variance in memory performance, *F*(1, 39) = 21.27, *p* < .001, *R*^2^ = 0.35. The second model, which included tobacco use, accounted for 42 % of the observed variance, *F*(1, 39) = 13.98, p < .001, *R*^2^ = 0.42 and resulted in a significant *R*^2^ change of 0.07, p = .04. | Comprehensive cohort – due to small numbers of exposed children and compulsory treatment in Norway. Direct assessment through validated task. | Smoking was major confounder with few unexposed children exposed to tobacco. |
| 28 | Sarfi, 2022, Children born to women in opioid maintenance treatment: A longitudinal study of child behavioral problems and parenting stress | This study aimed to describe (a) parenting stress, (b) perceived child behavioural problems, and (c) post-natal mental health of mothers in OMT and a comparison group of non-dependent mothers from infancy to early school age and to explore the association between child behaviour problems and parenting stress in the two groups across time | Norway | OMT - Buprenorphine or Methadone | Same as #2613. Follow-up at 8 years: OMT group = 26 participants; Controls = 24 participants. | Same as #2613 | Strengths and Difficulties Questionnaire – completed by mothers at age 8. | **Internalising symptoms and externalising behaviours:** SDQ total difficulties scores were higher for children in OMT group, but with substantial overlap between groups: OMT 5.9 (4.0); Controls 8.7(5.1). There was a strong positive correlation between parenting stress and child behaviour problems among mothers in OMT and a somewhat weaker association in the comparison group. | Longitudinal design. Rich data available. Over 66% retention rate. Minimal on-top use of illicit drugs due to close follow-up systems in Norway. | Small sample from single country therefore limited statistical power. Only one source of SDQ data – validation studies should greater predictive value with multiple respondents. |
| 29 | Sarfi, 2022, Mental health and use of health care services in opioid-exposed school-aged children compared to foster children | This study aimed to explore the distribution and level of different mental health problems among opioid-exposed children living with biological parent(s) or in foster care compared to those of non-opioid-exposed foster children. (i) As a secondary aim, to assess possible differences between teacher and caregiver reports, regarding mental health profiles for the three groups. 2. To describe service utilization among opioid-exposed children compared to foster children. (i) A secondary aim is to investigate the association between mental health problems and service utilisation, adjusted for group affiliation. | Norway | OMT - Buprenorphine or Methadone | This study brought together two samples from the same country: one sample was the same as #2613: 36 children born in 2005/6 and followup up at 2013-4, when children had reached schooled age (>6); the other sample comprised 44 children born in 2004. 2007. 2008 and 2009, with follow-up in 2016-17 – all children except those born in 2004 were age 6-8 when included in the study. Merged data provided data on 78 opioid exposed children. A comparison group of non-drug exposed children in foster care and aged 6-12 from a larger study of foster children (n=140). | 2 x birth cohort studies. Latest follow-up by telephone interview. | The telephone interview collected data on services used both for mothers and for the child. Services were reported as “did you ever have contact with”: CWS, education, child mental health, or habilitation services and “do you have ongoing contact with” the same services. Mental health data were collected from caregivers and the children’s teachers independently, through a secure online questionnaire (the Developmental and Wellbeing Assessment (DAWBA). SDQ was completed by parents. | **Removal from birth parents**: 37% of OMT children living with parents currently had experienced 1+ placements away from home.  **Internalising symptoms and externalising behaviours:**: For caregiver rated outcomes, the OMT home group had lower rates of mental health problems both compared to the OMT foster care group and the Foster care group as reflected by significant lower scores on emotion, conduct, hyperactivity/inattention, and peer problem subscales. For prosocial functioning in children living at home had significantly higher scores than the non-exposed foster care group, but not the OMT foster care children. Impact score, indicating perceived impairment in everyday functioning, was significantly lower in the OMT home group compared to non-exposed foster care children. There were no significant contrasts between the two foster care samples (OMT and non-OMT). In terms of teacher-rated SDQ, group-differences were only found for total difficulties, conduct problems and hyperactivity/inattention scales.  Inhibitied (RAD) and socially indiscriminate (DSED) behaviour: the OMT home group had lower levels of attachment difficulties symptoms, compared to both foster care groups.  **Service use:** 47% of caregivers in the OMT home group reported no use of any services for their children, compared to 19% of the OMT foster care group. In the Foster care group, the reported percent service use was similar to the OMT Foster group (no service use: 24% and one or more services: 59%). The authors note though that missing data were higher in reports from foster parents (OMT foster care 31.3% and Foster care group 16.4%) compared to biological caregivers (OMT home=3%). | High participation rate. Nationally representative sample of parents on OMT and children. Multi-informant assessments. | Sample sizes were small – especially in OMT foster care group. Author notes potential for Hawthorne effect biasing results. |
| 49 | VandenBosch, 2022, Neonatal Pain, Opioid, and Anesthetic Exposure; What Remains in the Human Brain After the Wheels of Time? | This paper aimed to evaluate possible negative long-term effects of neonatal exposure to pain, opioids and anaesthetics in children and adolescents | The Netherlands | NAS due to prenatal exposure to opioid related substances (morphine, methadone, heroin) taken as drugs of abuse by their mothers. | We extract results from this paper for the NAS cohort and their controls only (4 other cohorts were available). Children in an electronic patient system who were admitted to the Erasmus MC-Sophia Children’s Hospital in Rotterdam from October 1993 to May 2005 for treatment of NAS due to prenatal exposure to opioid related substances (morphine, methadone, heroin) taken as drugs of abuse by their mothers were recruited for the cohort and followed up with children aged at least 8 years old at follow-up (n.15). Controls were recruited through local primary schools. Inclusion criteria were term born children without a history of admission to a (Neonatal) Intensive Care Unit (NICU) and without neonatal opioid exposure or significant pain experience, analgesic or sedative use in the first year of life. Children were matched at a comparable age to the cohort (n.71). | Cohort study | Neuropsychological Assessment: The NEPSY-II-NL neuropsychological test was conducted in children up till 16 years of age. Children 8-12 years completed 9 subtests (attention and executive functioning, language, memory and learning, sensorimotor functioning, and visuospatial processing). Older participants performed only 6 of these subtests due to the age limit of the 3 other tests.  Detection and Pain Thresholds: Thermal Sensory Analyzer-II (e.g. reducing temperature and asking child to state when it felt cold/warm.  MRI: MR images were acquired on a 3 Tesla scanner  fMRI: functional MRI scans whilst performing detection and pain threshold tests. | **Cognitive development:** Neuropsychological Assessment: Children with NAS performed statistically significantly worse on several domains including visiospatial, language, attention and executive functioning tests compared to controls (Geometric Puzzles p = 0.002; Response Set (more omission errors) p = 0.002, Word Generation p = 0.002, and Arrows p = 0.002).  **Brain development:** Structural imaging: No differences were found in brain morphology between children with or without NAS.  fMRI: statistically significantly greater brain activation in one cluster consisting mainly of the frontal pole in the control group compared to the cases, but the significance disappeared after correction for age and gender.  **Pain:** Detection and Pain Thresholds: No differences were found in pain or detection thresholds between children with or without NAS. | Specific cohorts of exposure used with information of additional substance use available. Standardised assessment in clinical settings of children. | Sample size was very small and thus underpowered, and ages potentially ranged from 8-19, although this was unable to be verified for this particular cohort. Almost all children had other substance exposure as well as opioids, although 13/15 were exposed to methadone and 12/15 were exposed to heroin. Controls were asked to participate and were likely non-random, so selection bias is a possibility. Moreover, possible selection bias has occurred because children with the most severe neurological and cognitive outcomes did not participate. No attempt was made to control for confounding. |

**Supplemental Table 3 ROBINS-E Tool for assessing risk of bias among observational studies of exposure**

| **Ref** | **Author** | **Bias due to confounding** | **Bias due to measurement error** | **Bias in selection to study** | **Bias due to post-exposure interventions** | **Bias due to missing data** | **Bias in measurement of outcomes** | **Bias in selection of the reported result** | **Overall judgment** |
| --- | --- | --- | --- | --- | --- | --- | --- | --- | --- |
| 21 | Aslaksen, 2024a | High | Low | Low | Low | Low | Low | Low | Low |
| 23 | Aslaksen, 2024b | High | Low | Low | Low | Low | Low | Low | Low |
| 22 | Aslaksen, 2025 | High | Low | Low | Low | Very High | Low | Low | High |
| 46 | Auger, 2020a | High | Low | Low | No | Some concerns | Low | Low | Some concerns |
| 47 | Auger, 2020b | High | Low | Low | No | Some concerns | Low | Low | Some concerns |
| 32 | Azuine, 2019 | High | Very High | High | Low | High | High | Low | Very high |
| 33 | Bauer, 2020 | High | Low | High | Low | Low | Some concerns | Low | High |
| 34 | Miller, 2020a | High | Low | Some concerns | Low | High | Some concerns | Low | High |
| 35 | Miller, 2020b | High | Low | Some concerns | Low | High | Some concerns | Low | High |
| 24 | Konijnenberg, 2018 | High | Low | Low | Low | Low | Low | Low | Low |
| 25 | Konijnenberg, 2020 | High | Low | Low | Low | Some concerns | Low | Low | Some concerns |
| 26 | Konijnenberg, 2021 | Some concerns | Low | Low | Low | High | Low | Low | High |
| 27 | Konijnenberg, 2022 | High | Low | Low | Low | Very high | Low | Low | Very high |
| 28 | Sarfi, 2022a | High | Low | Low | Low | Very high | Low | Low | Very high |
| 29 | Sarfi, 2022b | High | Low | Low | Low | Very high | Low | Low | Very High |
| 36 | Corr, 2021 | Low | Low | Low | Low | Low | Low | Low | Low |
| 48 | Hamilton, 2024 | High | Low | Low | Low | Low | High | Low | High |
| 43 | Jaekel, 2021 | Very high | Low | Low | Low | Low | Low | Low | Low |
| 44 | Lee, 2019 | Low | Low | Low | Low | Low | Low | Low | Low |
| 45 | Newbury, 2024 | High | Low | Low | Low | Low | Low | Low | Low |
| 37 | Lambert, 2024 | High | Low | Low | High | Some concerns | Low | Low | High |
| 39 | Lee, 2024 | Some concerns | Low | Low | High | Low | Low | Low | High |
| 40 | Oei, 2017 | Low | Low | Low | Low | High | Low | Low | High |
| 41 | Uebel, 2015 | Low | Low | Low | Low | Low | Low | Low | Low |
| 42 | Uebel, 2024 | Low | Low | Low | Low | Low | Low | Low | Low |
| 50 | Lin, 2023 | High | Low | High | High | Low | Low | Low | High |
| 51 | Ornoy, 2016 | High | Low | Low | Low | Low | Low | Low | Low |
| 38 | Liu, 2019 | High | Low | Some concerns | High | Some concerns | Low | Low | High |
| 52 | Odsbu, 2023 | Low | Low | Low | Low | Low | Low | Low | Low |
| 30 | Sirnes, 2017 | Some concerns | Low | Some concerns | Low | Low | Low | Low | Some concerns |
| 31 | Sirnes, 2018 | High | Low | Some concerns | Low | High | Low | Low | High |
| 49 | VandenBosch, 2022 | Very high* | - | - | - | - | - | - | Very high |

Abbreviations: NI: no information, ROBINS-E: risk of bias in non-randomized studies of exposures The risk of bias in non-randomized studies of exposures (ROBINS-E) tool comprises 7 domains through which bias might be introduced. The questions of these domains include: (1) bias due to confounding, (2) bias in selection of participants into study, (3) bias in selection to study, (4) bias due to post-exposure interventions, (5) bias due to missing data, (6) bias in the measurement of outcomes, and (7) bias in the selection of reported results. In addition, the final column presents a summary variable of overall risk of bias. Studies were categorized as Low risk of bias, Some concerns, High risk of bias, or Very high risk of bias, under each domain. In the summary variable, Low risk of bias may contain an exception for concerns about residual confounding (“Low risk of bias except for concerns about residual confounding”). *Note, where results are purely descriptive and confounders are not controlled for, this is deemed to be at Very High risk of bias and no further analysis is undertaken.
